# Laser irradiation of human skin tissue after gold nanoparticles injection for thermal ablation processes – a combined experimental and numerical approach

**DOI:** 10.1038/s41598-025-17459-3

**Published:** 2025-10-02

**Authors:** Marta Cecotka, Piotr Radomski, Paweł Ziółkowski, Agata Tymińska, Katarzyna Czerwiec, Jacek Zieliński, Yue Dong, Christian Rossner, Francesca Petronella, Magdalena Narajczyk, Jakub Karczewski, Luciano De Sio, Michał Pikuła, Dariusz Mikielewicz

**Affiliations:** 1 Faculty of Mechanical Engineering and Ship Technology, Gdańsk University of Technology, Institute of Energy, Narutowicza 11/12, 80-233 Gdańsk, Poland; 2 Laboratory of Tissue Engineering and Regenerative Medicine, Division of Embryology, Department of Anatomy, Faculty of Medicine, Medical University of Gdańsk, Gdańsk, Poland; 3 Laboratory of Tissue Engineering and Regenerative Medicine, Division of Clinical Anatomy, Department of Anatomy, Faculty of Medicine, Medical University of Gdańsk, Gdańsk, Poland; 4 Department of Oncologic Surger, Medical University of Gdańsk, Gdańsk, Poland; 5 Leibniz-Institut für Polymerforschung Dresden e.V., Hohe Straße 6, 01069 Dresden, Germany; 6 Faculty of Chemistry and Food Chemistry, Technische Universität Dresden, 01069 Dresden, Germany; 7 Department of Polymers, University of Chemistry and Technology Prague, Technická 5, Prague 6, 166 28 Czech Republic; 8 National Research Council of Italy, Institute of Crystallography CNR-IC, Montelibretti Division, Area Territoriale di Ricerca di Roma 1 Strada Provinciale 35d, Montelibretti, 00010 Italy; 9 Faculty of Biology, Bioimaging Laboratory, University of Gdańsk, Gdańsk, Poland; 10 Faculty of Applied Physics and Mathematics, Gdańsk University of Technology, Institute of Nanotechnology and Materials Engineering, Narutowicza 11/12, 80-233 Gdańsk, Poland; 11 Department of Medico-Surgical Sciences and Biotechnologies, Sapienza University of Rome, Corso della Repubblica 79, 04100 Latina, Italy

**Keywords:** Human skin, Laser irradiation, Gold nanoparticles, Photo-thermal ablation, Computational models, Nanoparticles, Nanowires, Lasers, LEDs and light sources, Biomedical engineering, Fluid dynamics, Targeted therapies

## Abstract

In recent years, investigation of new or combined therapeutic modalities for cancer treatment lead to relevant scientific advances. Among these innovative therapeutic modalities photothermal therapy (PTT) attracts attention as an alternative or complementary possibility to current anti-cancer treatments as it allows for the selective ablation of cancer cells. In the present study, PTT is investigated by using gold nanoparticles (AuNPs) as photothermal transducers. AuNPs are injected into human skin and used for PTT-assisted tumor ablation. The effectiveness of the PTT is evaluated as a function of AuNPs morphology, dimensions and the wavelength of the laser source. Five different laser sources with wavelengths ranging from 465 to 980 nm are used. The experimental findings point out that 808 and 980 nm are the optimal laser wavelengths for the PTT of human skin. Especially gold nanorods stabilized by poly(ethylene glycol) layer are identified as effective photothermal transducers. The experimental results are fully corroborated by numerical calculations. The outcomes emphasize the potential and the relevance of synthetically tailoring AuNPs particularly in optimizing their optical properties and assessing the effects of AuNP aggregation for specific applications.

## Introduction

Cancer, as an aggressive and fatal disease, is one of the greatest global health concerns as its cases of incidence are considerably growing. Despite significant progress in cancer treatment, it still remains one of the most common causes of death worldwide. Europe faces a significantly higher burden of cancer incidence and mortality compared to its share of the global population. Despite accounting for less than 10% of the world’s population, Europeans experience 22.4% (almost 4.5 million) of global cancer causes and 20.4% of cancer-related deaths (data for the year 2022)^1^. Approximately one in five individuals is diagnosed with cancer at some point in his life, while one in nine men and one in twelve women lose their lives to the lethality of the disease^1^. Traditional cancer treatments (e.g. surgery, chemotherapy, radiation therapy) often cause severe side effects, damaging healthy tissues and significantly reducing patients’ quality of life^2^. Hence, there is an abiding need for a treatment method that, preferably in a non-invasive way, will eliminate adverse cancer cells^3^. To lay the groundwork for advancing into skin cancer research, promising preliminary tests were conducted on healthy skin samples using lasers of various wavelengths and incorporating nanoparticles.

### Human skin – fundamentals

**Fig. 1:**
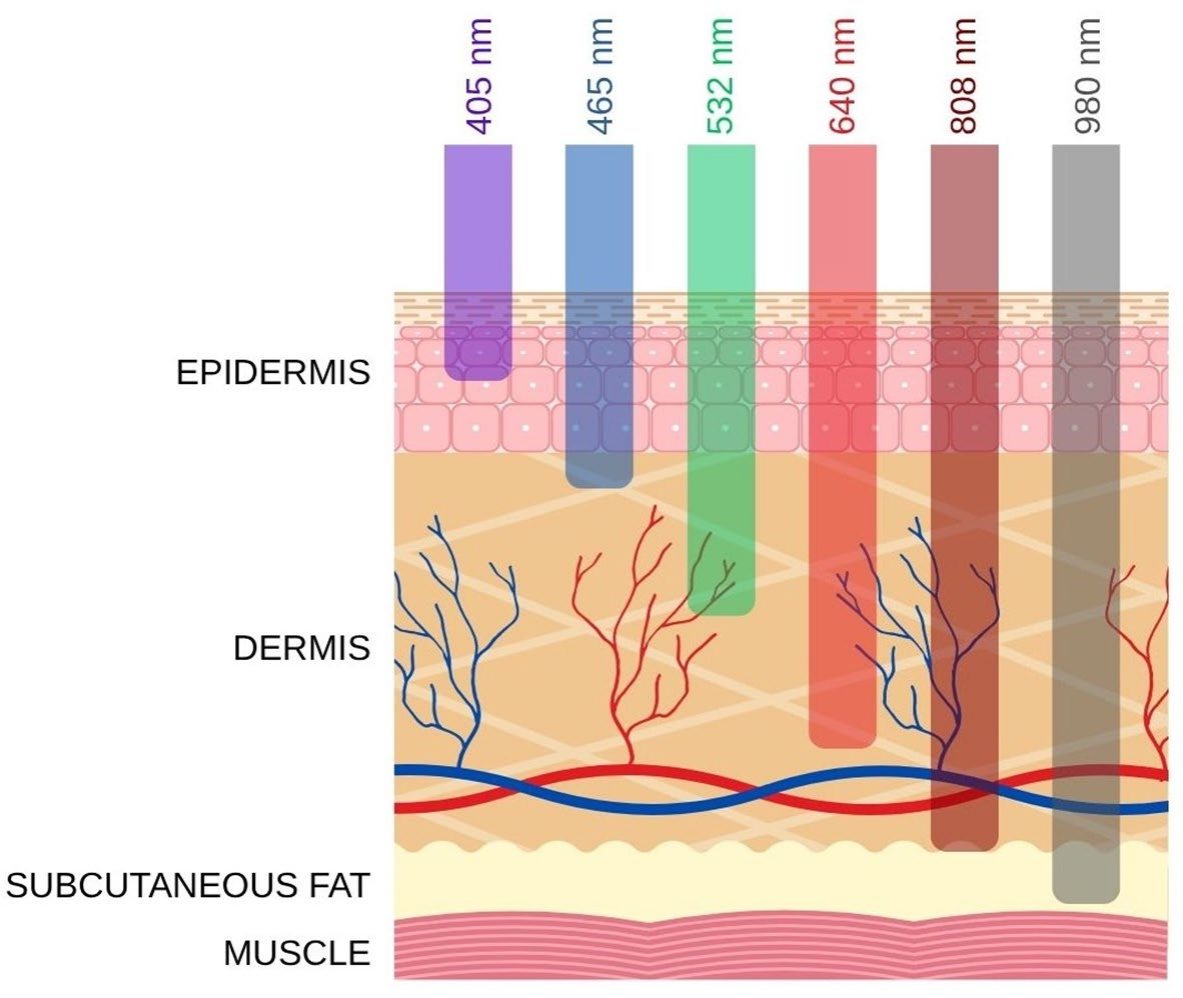
Dermal penetration of different wavelengths of light^6^.

Three layers are generally distinguished to human skin – epidermis, dermis and subcutaneous tissue (Fig. 1). The epidermis is the outermost layer of the skin and is made up of keratinocytes. It also contains melanocytes that produce the skin pigment, melanin. Next is the dermis, composed of fibroblasts, which also contains blood vessels, hair follicles, sweat glands and nerves. The deepest layer is subcutaneous tissue, which, in addition to collagen and the proteoglycan matrix, includes fat cells, namely the adipocytes. Human skin, as the largest organ of the body, provides a primary protective function against mechanical, physical, chemical, and microbiological factors. It also plays a role in the synthesis (e.g. vitamin D), and metabolism of proteins, lipids and carbohydrates^4^. Due to the great variety of cells that make up the skin, there are many different types of skin cancers. However, the two most significant groups are melanoma skin cancers (MSC) and non-melanoma skin cancers, such as basal cell or squamous cell carcinoma. In Europe, MSC is the sixth most frequently diagnosed cancer with 10–25 new cases per 100,000 inhabitants (data for 2020)^5^.

### Hyperthermia as a method of destroying cancerous cells

Hyperthermia has been widely used for many years as an effective method for destroying cancerous mass lesions. Its use dates back over 4000 years, when even the molecular basis of cancer was unknown and accurate diagnoses were not possible. Reasons why hyperthermia is so effective in destroying malignant cancerous cells are few. Pre-eminently, tumor cells often have inadequate blood supply, which reduces their thermoregulation ability. As a result, they heat much more than healthy tissues, especially when local heat generation is aimed. Furthermore, tumor lesions frequently contain hypoxic cells, with low pH or with nutrient deficiency. These conditions collectively increase the sensitivity of malignant cells to heat-induced damage. Hyperthermia can be induced through various methods, including the RFA (Radio Frequency Ablation) technique, electrosurgery, ultrasounds, MWA (Microwave Ablation) or emerging approaches – targeted hyperthermia induced by gold or gold-based nanoparticles, as well as irreversible electroporation (IRE)^7–9^.

### Why gold nanoparticles?

Gold nanoparticles are highly valued in many applications, including biomedicine, due to their unique property known as a Localized Surface Plasmon Resonance (LSPR)^10,11^. This phenomenon occurs as a result of the collective oscillation of surface electrons (plasmons) when nanoparticles interact with light of a resonant wavelength, referred to as the plasmon resonance wavelength. This resonant excitation creates a pronounced peak in the absorption spectrum, known as the plasmon peak. For gold nanoparticles, the spectral position and magnitude of this resonance can be engineered by adjusting the nanoparticle’s dimension and shape^12^. This allows to tune the resonance wavelength from visible frequency into the NIR region, especially for gold nanorods. The NIR region is further divided into two tissue transparency windows: the first NIR window (650–850 nm) and the second NIR window (950–1350 nm). In the second window, the absorption of radiation by biological tissues is minimal, allowing for deeper penetration, while minimizing adverse effects on surrounding healthy tissues. Consequently, nanoparticles designed to absorb within the second NIR window are suitable for treating tumors located deeper within tissues - up to approximately 100 mm - due to the enhanced penetration depth of radiation in this range. Conversely, radiation within the first NIR window is more effective for superficial solid tumors, where the penetration depth ranges between 20 and 30 mm. Due to the enhanced tissue transparency in these spectral regions, gold nanorods have been successfully applied as subcutaneous implantable biosensors^13^. The ability to precisely tune the absorption wavelength of nanoparticles is crucial in photothermal therapy, as it enhances therapeutic efficacy while minimizing collateral damage to surrounding healthy tissues^14,15^. Under LSPR conditions, light absorption is followed both radiative and non-radiative relaxation processes. Amongst the non-radiative processes, the most significant is plasmonic heating, which enables generation of intense, localized heating^16–18^.

Additionally, nanoparticles may enable targeting of cancerous tissue. The vascular system of tumor structures contains relatively large gaps (100 nm ÷ 2 μm), making them particularly permeable to nanoparticle penetration (EPR effect)^19,20^. Additionally, due to the absence of an efficient lymphatic drainage system, tumors are unable to remove these foreign structures effectively, leading to their accumulation within the tumor tissue^21^. A further crucial property of nanoparticles is their ability to have their surface modified with biomolecules to ensure biocompatibility and enable selective recognition of cancer cells (so they will not accumulate in both cancerous and healthy cells in case of distribution via human circulatory system)^22–24^.

Electromagnetic radiation interacts in a wide spectrum with virtually all organic and non-organic substances. Unlike white light from conventional sources, which is visible for human’s eye, laser beam is highly focused, coherent and monochromatic with a single wavelength and exceptionally high intensity, which can be converted into other forms of the useful energy.

For this reason, laser radiation is also regarded as a remarkable tool in material science as it can be used for preparing or modifying nanostructures. Laser ablation is used for preparing free standing electrodes^25^ modifying nanocomposites with metallic nanoparticles^26,27^. The irradiation of gold nanoparticles in combination with silver offers prospects in more branches of medicine, as they show satisfactory antibacterial behavior with a ratio of 2/3 Au/Ag^28^. In addition, silver/gold tuning into core-shell structures has been demonstrated to provide support for wound healing by laser ablation^29^. In addition, it has been proven that thermal stability is increased by the addition of Au NPs^25^, which improves the functionalization of both medical processes and technical devices.

Laser sources are extensively used for investigating light-matter interactions, especially for plasmonic nanomaterials. Hence, having tuned the incident wavelength to the oscillation frequency of plasmons, the LSPR can be triggered by laser irradiation providing the effective heating of irradiated nanoparticles and boosting temperature rise, which could lead to faster decay of tumors^30,31^.

Several parameters define laser irradiation, with wavelength being the most critical, as it determines the depth of light penetration into tissues (longer wavelengths correspond to deeper penetration). Other key parameters include the energy density and the duration of exposure, both of which determine the total irradiation dose absorbed by cells. Furthermore, the nature of the laser beam – continuous or pulsed – also significantly affects its interaction with biological tissues^6^.

Another crucial aspect to consider is the behavior of the laser beam after penetrating human skin. The primary processes involved are absorption and scattering. A portion of the radiation will be reflected (researchers estimate that 4–7% of visible light is reflected from the skin surface) or transmitted through the tissue. Chromophores, i.e. compounds that absorb light at a particular wavelength, are responsible for light absorption. In the skin, the primary (visible and NIR) chromophores are hemoglobin, melanin and water. Scattering is mainly caused by filamentous proteins such as keratin in the epidermis and collagen in the dermis. The extent of scattered laser energy is inversely proportional to the wavelength, meaning that longer wavelengths scatter less, allowing for deeper tissue penetration. In the NIR, water becomes the dominant absorber of radiation. A fundamental challenge in modeling or analyzing optical phenomena in biological tissues, particularly skin, lies in accurately determining the absorption and scattering coefficients. This task is highly complex due to the numerous factors influencing these parameters, including age, skin pigmentation, layer thickness, individual physiology, tissue hydration levels, and genetic variation^6,32^.

### Tissue modeling – numerical approach

Advances in PTT depend on sophisticated biological heat transfer models that simulate precise thermal dynamics within tissues. The fundamental Pennes equation, known for its simple representation of thermal conduction and perfusion heat transfer in tissues, provides the basis for more complex models^33,34^. Based onthis, the Weinbaum-Jiji model incorporates detailed consideration of vascular geometry^35^, providing a more profound understanding of the localized thermal effects of blood flow, which is essential to achieve precise thermal targeting in PTT. For scenarios that require high-resolution tissue heterogeneity, the porous media theory model treats tissues as porous structures to explore local thermal equilibrium and non-equilibrium conditions^36^, improving the scope of the model’s application to complex biological structures. Non-Fourier models, such as the Thermal Wave and Dual-Phase-Lag^35^ treat the rapid thermal transitions induced by laser treatments, capturing the late thermal response characteristics essential for optimizing laser pulse parameters^37^. These models provide a solid theoretical base for the development of advanced PTT strategies that exploit the unique interactions of light and heat in biological tissues, promising greater treatment precision and safety. In addition, the Arrhenius equation and first-order kinetics are essential for modelling protein denaturation and thermal damage in skin tissue subjected to PTT. These models help predict the temperature thresholds at which cellular proteins begin to denature, leading to cell damage or death, providing the quantitative insight needed to optimize treatment parameters to minimize unintended damage^37^. However, the essence of all these models is to take into account the correct mass, momentum and energy balances and to give material properties that correspond to the actual process parameters^38,39^. For this purpose, calibration is carried out both of the process of conversion of electromagnetic energy into heat itself^40^ and, at a later stage, of heat conduction in the body of the tissue or other material^41^.

### Research motivation and novelties of article

The motivation for this research stems from the potential to combine the unique properties of gold nanoparticles, the interaction of laser light with human skin cells, and the structural characteristics of cancerous tissues. The porous nature of tumors, the small size of nanoparticles, and the ability to modify their surface to selectively recognize and accumulate in cancer cells enable targeted distribution of nanoparticles specifically within malignant tissues, thereby minimizing impact on healthy tissues^42^. Furthermore, the existence of a ‘biological window’ in the NIR, the use of lasers with precise beam wavelengths, and the ability to manipulate the nanoparticles’ maximum radiation absorption range make it possible to optimize all system parameters. The optimization facilitates efficient heat generation within tissues, achieving the temperatures necessary for a destruction of harmful cells^43^.

Currently, there is a scarcity of literature on the application of gold nanoparticles in the thermal ablation of tumors within the context of human skin. This indicates a research gap and a growing demand for studies in this area. Previous publications have predominantly focused on laser-irradiated samples derived from cell lines that mimic human skin cells or on experiments conducted on animals, such as mice^44–46^. Additionally, numerous studies have explored computational models designed to approximate real-world conditions^47–49^.

In the previous studies by the authors, a satisfactory agreement was observed between the simulation results and the experimental data for both white-light lamps^10,39^, and for laser sources^39,41,50,51^, with respect to temperature distributions and temporal characteristics of gold nanorods deposited on a different substrates and located in simple working fluids. However, to date, no real biological tissue or structure has been taken into account in these investigations, especially in the system containing nanoparticles.

Hence, the specific goal of this work was to experimentally test the process of converting electromagnetic energy into heat for various conditions, including AuNP size, shape, and surface-functionalization as well as irradiation source; and to validate the obtained results by a theoretical model, for identifying the optimal parameters for the photothermal skin cancer treatments.

The present study advances the existing framework developed by the authors and integrates experimental investigations on human skin with the development of a computational model designed to estimate temperature increase and temperature distributions across individual skin layers, which would allow rapid and robust determination of these parameters within the tissue. The experiments and analyses conducted in this research were designed to identify the critical nanoparticle parameters and laser wavelengths, aiming to develop a therapy with the potential for minimum invasiveness to healthy cells, while effectively overheating pathogenic cells.

Moreover, these are the first known and documented results of laser-irradiated human skin where gold nanoparticles were directly injected into human skin. Reaching the critical temperature at which tumor cells undergo necrosis and metabolic activity ceases is key to establish the effectiveness of thermal ablation process. The application of nanoparticles that are capable of generating localized heating at the surface of tumor cells enhances the noninvasiveness of such procedures. In this context, numerical simulations, if the used model is valid, serve as a powerful and non-destructive tool for predicting temperature increase is a key parameter in assessing experimental conditions that ensure both the noninvasiveness and efficacy of the thermal ablation process. Thus, the obtained results represent an extremely relevant step both in the context of using nanoparticles as a component of the mixture injected into the human body and to provide an excellent basis for the development of numerical calculations using thermal ablation.

## Methods

### Measurement setup and protocol for the laser experimental part

**Fig. 2:**
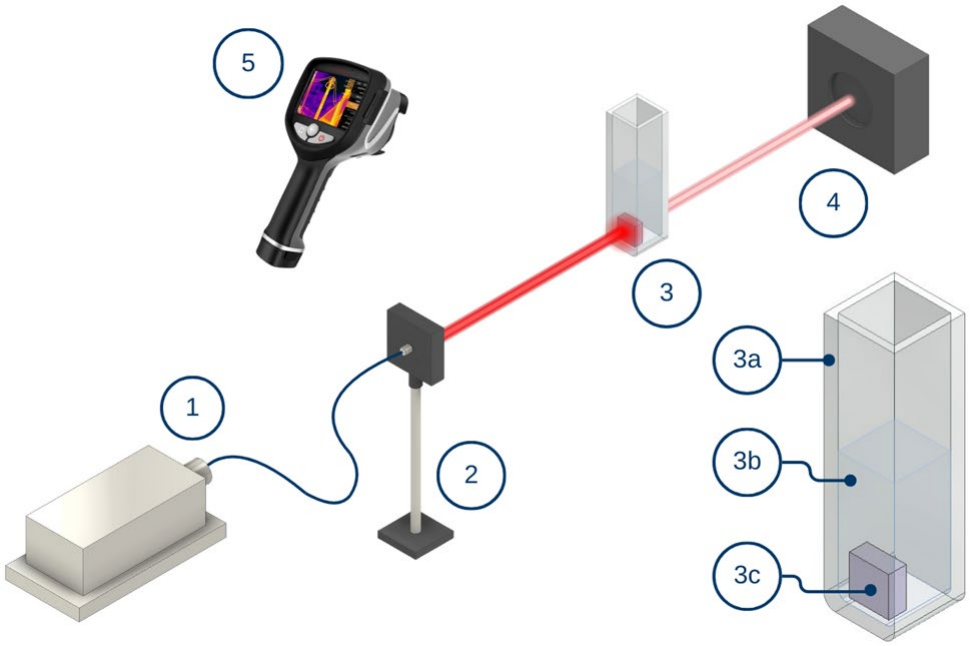
Measurement setup, where 1 – laser with selected wavelength, 2 – optical fiber holder, 3a – quartz cuvette, 3b – 2 ml of phosphate buffered saline (PBS), 3c – human skin sample (7 × 7 mm), 4 – laser power meter, 5 – thermal camera. 3D models of the measurement station elements were created using Autodesk Inventor Professional 2023 (https://www.autodesk.com/pl - student version), while their layout and descriptions were created in Canva (https://www.canva.com/).

The measurement set up included a laser, a quartz cuvette containing a skin sample, a laser power meter, and a thermal camera (Fig. 2). The protocol for the experiment was as follows: first, a piece of skin constituting surgical waste was collected from patients undergoing surgery. Medical waste was used for scientific research only after taking prior consent for the collection of a tissue fragment. Thus, informed consent was obtained from all subjects and/or their legal guardians before the aforementioned medical waste was used. Secondly, the sample was sectioned into smaller pieces with dimensions of 7 × 7 mm. Thirdly, these samples were exposed to laser irradiation in quartz cuvettes with 2 ml of PBS (Phosphate Buffered Saline from SIGMA-ALDRICH CHEMIE GmbH). The experimental procedure was approved by the Bioethics Commission for Research at the Medical University of Gdańsk (KB/121/2024 and KB/121–368/2024). The Commission issued a resolution granting a favorable opinion, as the study constitutes cognitive fundamental research that does not raise ethical concerns. All experiments and methods were conducted in accordance with relevant guidelines and regulations.

Experiments were divided into two phases. The first phase contains irradiating control samples for 10 min using five different laser wavelengths to evaluate their cytotoxic effect on human skin cells. In the second phase, gold nanoparticles were injected into skin fragments (in a volume of 15 µl), followed by irradiation with two selected laser wavelengths (808 and 980 nm), which were selected due to minimizing optical density of human skin (‘biological window’), supported by literature data^52–54^. The irradiation duration in this phase was either 1.5–5 min.

Data of three parameters were collected during experiments. To investigate thermal changes during laser exposure, temperature distributions and temporal responses were recorded on the front wall of the cuvette using an InfiRay T600 thermal camera (640 × 512 resolution, 24° × 18° field of view, 35 mK thermal sensitivity). The emissivity was calibrated for quartz glass at 0.93, while the environmental emissivity was set at 0.95. Initial temperature and humidity were set at 22.0 °C and 40% respectively. To analyze the power absorbed and transmitted by the system, a laser power meter, Gentech 11MAESTRO with detector 11UP19K-15 S-H5 from the Standa Ltd was used. Five continuous laser wavelengths (λ) were used: 465 nm (0.8 W, 2 mm beam size), 532 nm (1.1 W, 1.2 mm beam size), 640 nm (0.8 W, 2 mm beam size), 808 nm (0.8 W, 2 mm beam size) and 980 nm (0.8 W, 2 mm beam size). The 532 nm laser wavelength, with an elevated value of power density, was selected to enhance heat generation due to the fact that the transverse resonant peak of gold nanoparticles is localized close to this frequency. Quartz cuvettes with dimensions of 45 × 12.5 × 12.5 mm and 10 mm optical path length were purchased from CHEMLAND (product code: 11-Q-104).

**Fig. 3:**
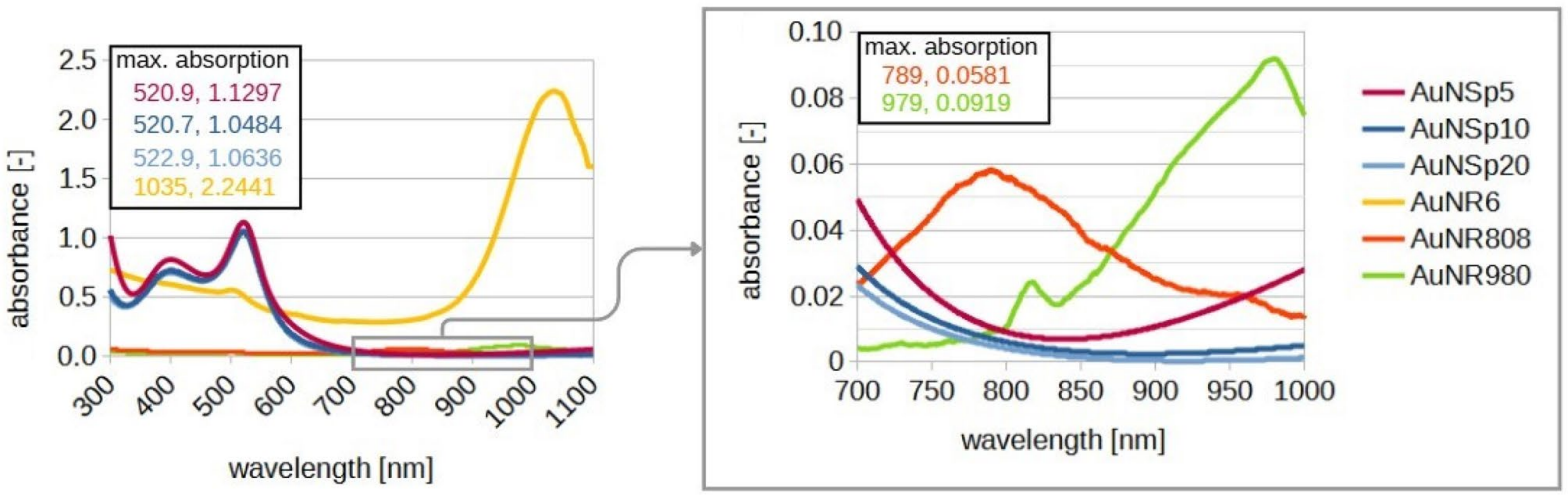
Absorption spectra of the used nanoparticles solutions of commercial gold nanospheres. (AuNSp5, AuNSp10, AuNSp20) and the own-made AuNR6 gold nanorods and commercial gold nanorods (AuNR808 and AuNR980).

**Fig. 4:**
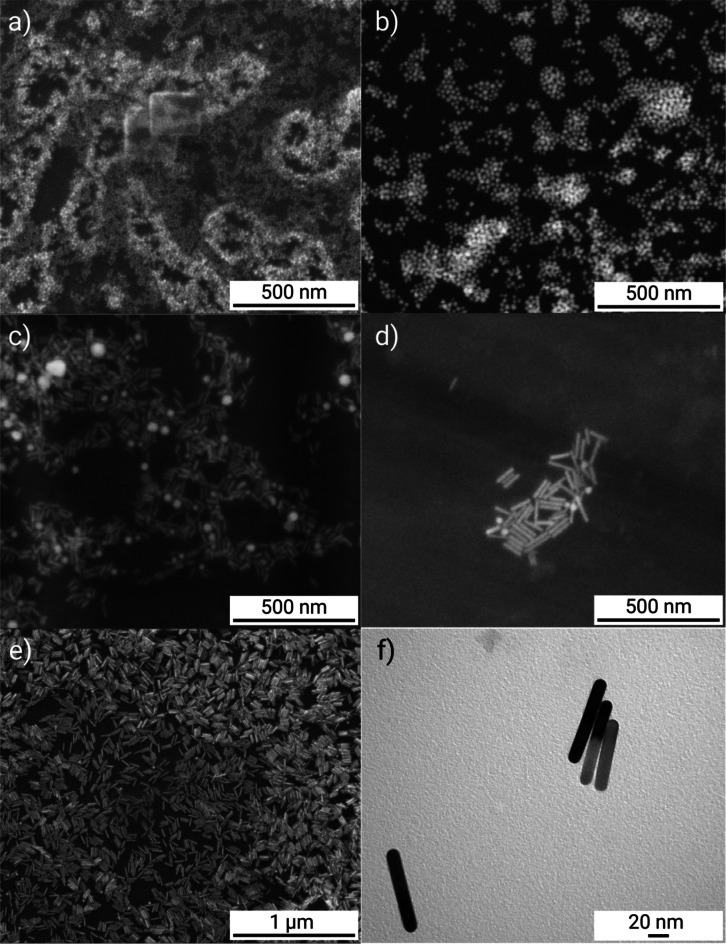
SEM images of: (**a**) AuNSp10, (**b**) AuNSp20, (**c**) AuNR808, (**d**) AuNR980, (**e**) AuNR6; (**f**) TEM image of AuNR6.

**Fig. 5:**
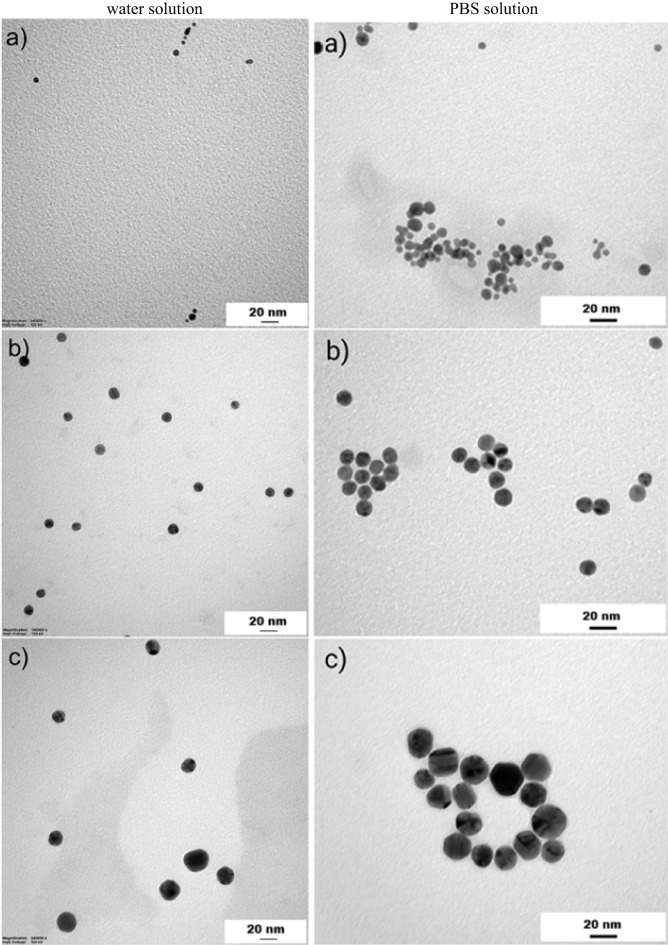

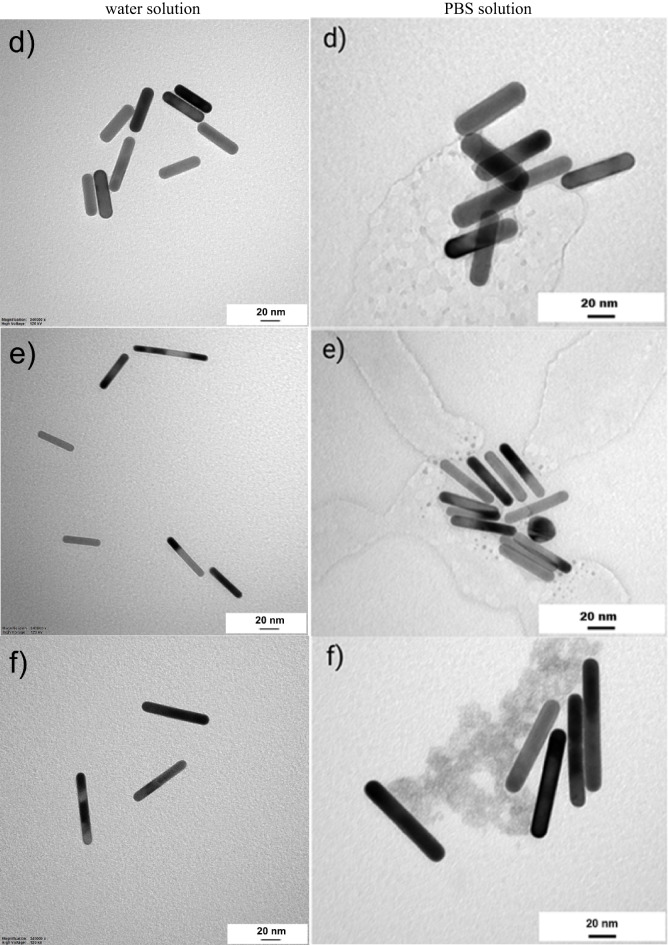
TEM images of: (**a**) AuNSp5, (**b**) AuNSp10, (**c**) AuNSp20, (**d**) AuNR808, (**e**) AuNR980, (**f**) AuNR6 dispersed in (left) water, (right) PBS solution what imitates the human skin conditions. Noticeably, nanoparticles exhibit a strong tendency to agglomerate and adhere to the PBS particles, which is considered to reflect the similarity of the human skin – gold nanoparticles interaction.

Solutions containing gold nanoparticles with different dimensions were used during the experimental part. Supporting research in literature, the commercial ones, AuNSp5, AuNSp10, AuNSp20, AuNR808, AuNR980, purchased from SIGMA-ALDRICH CHEMIE GmbH, had been stabilized and capped in citrate buffer. The absorption spectra of the used nanoparticles solutions and SEM images are shown in Figs. 3 and 4, respectively. The intention was to possess approximately the same value of mass concentration. However, as experimental studies have shown mass concentration does not play a decisive role, on the thermal effect because the main factor is the agglomeration of nanoparticles. As a confirmation of the occurring process under the influence of an environment close to biological conditions, nanoparticles from PBS were tested and the agglomeration effect is presented in Fig. 5 (PBS solution).

The other sample, named AuNR6, was synthesized by the seed-assisted binary surfactant method and capped by poly(ethylene glycol) (PEG) by subsequent ligand exchange process^55^. The gold seeds were created by mixing 5 ml of 0.5 mM HAuCl_4_ with 5 ml of 0.2 M CTAB followed by adding 0.3 ml of 20 mM NaBH_4_ under string. The mixture was further aged for 1 h at room temperature. For tuning the dimension of the gold nanorods to have the LSPR locate at the second NIR window, 7 g of CTAB, 1.234 g of Sodium Oleate (NaOL, Sigma-Aldrich, ≥ 99%)) were mixed with 250 ml water under stirring until dissolution. The mixture was set to 30 ℃ and added 18 ml of 4 mM AgNO_3_ (Sigma-Aldrich, ≥ 99%). Then, 250 ml of 1 mM HAuCl_4_ were added into the mixture and kept stirring for 90 min until the mixture became colorless. 3 ml (32%) of HCl were then added to adjust the pH followed by stirring for 10 min. 1.25 ml freshly prepared 0.1 M L-ascorbic acid (Sigma-Aldrich, reagent grade) were added to the mixture under string. Finally, 0.2 ml gold seed solution were added, and the final mixture was left undisturbed for 12 h at 30 °C. The solution obtained was then washed by water for 2 times and 1 mM CTAB solution for 1 time. To cap the Au NRs with PEG, 100 µl of 20 mg/ml Thiol-functionalized PEG (average M_n_ 6,000, Sigma-Aldrich) was added to concentrated Au NRs solution (1 mg/ml of Au [0]) under stirring. Then, 1 ml of methanol was added into the solution to assist the completely ligand exchange. The solution was stirred overnight and then washed by water for 3 times.

The final mass concentration of the prepared sample was intentionally about one rank higher to investigate the influence of the mass concentration in comparison to the commercial one, AuNR980nm, whose longitudinal resonant peak position was assumed to be located close to 980 nm. To establish spectral absorbance, and to confirm localized surface plasmonic bands of the used nanorods, a Thermo Fisher Scientific Evolution 220 spectrophotometer was selected. Spectra had been earlier calibrated to a quartz cuvette filled with 2-ml deionized water and were measured in the transmission mode at the spectral range from 300 to 1100 nm with a 1-nm step precision. Figure 3 presents spectra of the used nanoparticles.

Table 1 highlights dimensions and properties of the used nanoparticles.

**Table 1:** Properties of nanoparticles injected into skin fragments during experiments.

Symbol	Nanoparticles type	Dimensions [nm]	Max absorption wavelength [nm]	Mass concentration [µg/ml]	Manufacturer
AuNSp5	gold nanospheres	⌀5 (core size: 4–7)	520	69.4	code: 741,949 SIGMA-ALDRICH CHEMIE GmbH
AuNSp10	gold nanospheres	⌀10 (core size: 8–12)	520	60.7	code: 741,957 SIGMA-ALDRICH CHEMIE GmbH
AuNSp20	gold nanospheres	⌀20 (core size: 18–22)	520	53.1	code: 741,965 SIGMA-ALDRICH CHEMIE GmbH
AuNR808	gold nanorods	⌀10 ± 2 length: 39–43	808	35.0–45.0	code: 900,363 SIGMA-ALDRICH CHEMIE GmbH
AuNR980	gold nanorods	⌀10 ± 2 length: 57–61	980	35.0	code: 900,364 SIGMA-ALDRICH CHEMIE GmbH
AuNR6	gold nanorods	⌀15 ± 2 length: 85–91	1020	322.0	Own, see methods part

The AuNRs morphology was analyzed with an SEM FEI Quanta FEG 250. The first technique considers structures at their initial concentration which they were deposited on the etched silicon base wafers, operating at 0.003 bar, and measured under 10–20 kV voltage. Data were collected using an ETD detector forsecondary electrons and at either 50,000X or 100,000X magnification using a spot size 3. For TEM analysis, samples in two solutions, i.e., water and PBS, were prepared at the same concentration for the corresponding nanoparticles and subsequently were adsorbed onto 300 mesh grids (Agar) coated with 2% collodion (Merck) and carbon. Nanoparticles both in water and PBS were analyzed at the same working conditions using a Tecnai Spirit BioTwin transmission electron microscope at accelerated voltage of 120 kV. Images of the used samples were demonstrated in Figs. 4 and 5, respectively.

### Elementary CFD model of the system

**Fig. 6:**
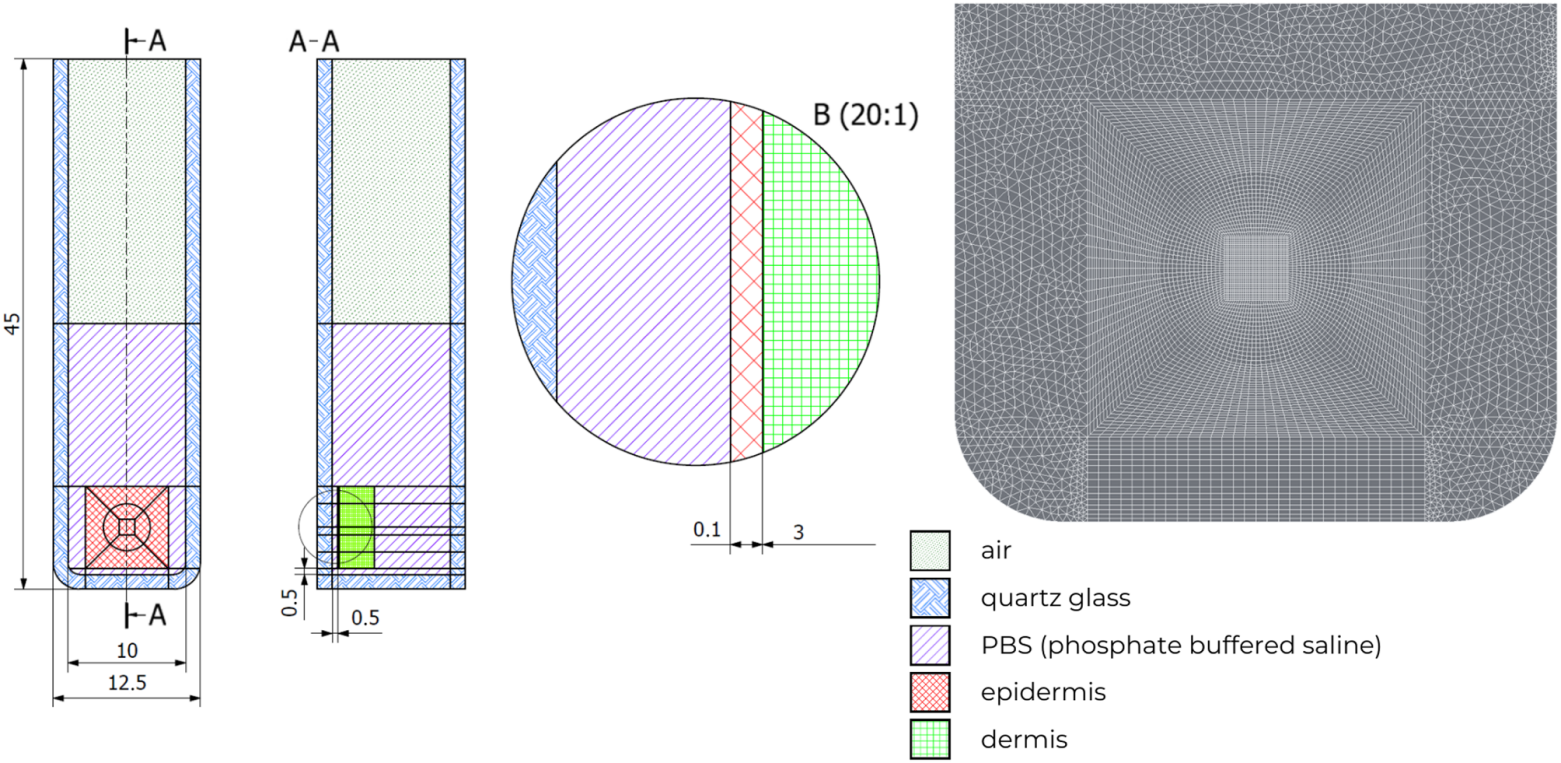
A schematic representation of the geometry used in the model, including the individual materials, and used mesh.

Due to the sensitivity of skin samples, it has been realized that it is impossible to carry out measurements of skin temperature in a non-destructive manner, including with a thermal imaging camera or thermocouple. To destroy cancerous cells, there is a need to obtain a temperature in a range of 52–60 °C. Taking into account body temperature, which is 36.6 °C, a minimal 16-degree temperature rise is expected to rule the process as effective. However, it was possible to obtain temperature data only for the front wall of the cuvette with the current measuring setup. Introduction of a model is a key to estimate temperature inside tissue and also for each individual layer. In Fig. 6, the used geometry is schematically presented, while Table 2 summarizes the key parameters of the individual components. The model takes into account a quartz glass cuvette, an air layer, and PBS in which the human skin sample was immersed and which reflects the environment in the human body. The provided tissue fragments were devoid of the subcutaneous fat layer; therefore, only the epidermis and dermis were modeled. The absorption and scattering coefficient values for the mentioned skin layers were taken from the literature, as well as refractive indices for PBS, dermis and epidermis. In this study, the anisotropy factor was assumed to be zero, based on the experimental setup in which the laser beam was directed perpendicularly to the surface of the human skin sample. As a result, the reduced scattering coefficient, which is commonly used in tissue modeling, was considered equivalent to the isotropic scattering coefficient^53,56,57^.

**Table 2:** Parameters of the quartz glass, air, PBS and individual skin layers used in the model.

		Model I – data from literature		Model II – assumed values	
		Laser wavelength [nm]		Laser wavelength [nm]	
		λ=808	λ=980	λ=808	λ=980
P_0_	[W]	0.8	0.8	0.8	0.8
n_cuv_	[-]	1.4532	1.4507	1.4532	1.4507
n_air_	[-]	1.000275	1.00027423	1.000275	1.00027423
n_PBS_	[-]	1.3336	1.341536	1.3336	1.341536
n_ep_	[-]	1.41737	1.379537	1.41737	1.379537
n_der_	[-]	1.38437	1.379537	1.38437	1.379537
µ_a ep_	[1/m]	171.035	45.035	513.0	135.0
µ_a der_	[1/m]	122.035	79.035	366.0	237.0
µ_s ep_	[1/m]	3680.035	3060.035	3680.035	3060.035
µ_s der_	[1/m]	2250.035	1860.035	2250.035	1860.035
D_ep_	[m]	0.0001	0.0001	0.0001	0.0001
D_der_	[m]	0.003	0.003	0.003	0.003

where:

P_0_– laser power;

n– refractive index;

µ_a_– absorption coefficient;

µ_s_– scattering coefficient;

D– dimension of the layer (thickness);

cuv– a quartz cuvette;

der– dermis;

ep– epidermis;

PBS– Phosphate Buffered Saline.

Firstly, the model is based on three fundamental CFD equations, namely: the mass conservation equation, the momentum conservation equation, and the energy conservation equation. Particular attention was given to the energy conservation equation. Secondly, the heat generated by the laser in each layer of the model (epidermis, dermis, PBS) was considered by calculating the average power density of the laser beam (due to the Gaussian distribution) and accounting for dimensions – the beam diameter and layer thickness. The absorbed, reflected, and scattered laser energy in each layer is calculated. The respective formulas are provided below.

Fundamental computational fluid dynamics (CFD) equations^58^:

1 $$\:\frac{\partial\:}{\partial\:t}\left\{\genfrac{}{}{0pt}{}{\rho\:}{\begin{array}{c}\rho\:\varvec{v}\:\\\:\rho\:e\end{array}}\right\}+\text{div}\left\{\genfrac{}{}{0pt}{}{\rho\:\varvec{v}\:}{\begin{array}{c}\rho\:\varvec{v}\:\otimes  \varvec{v}\:+p\varvec{I}\\\:\rho\:e\varvec{v}\:+p\varvec{v}\:\end{array}}\right\}=\text{div}\left\{\genfrac{}{}{0pt}{}{0}{\begin{array}{c}\varvec{\tau}\:\\\:\varvec{\tau}\:\varvec{v}\:+k\cdot grad\left(T\right)\:\end{array}}\right\}+\left\{\genfrac{}{}{0pt}{}{0}{\begin{array}{c}\left(\rho\:-{\rho\:}_{o}\right)\cdot \varvec{g}\\\:{\dot{q}}_{\text{A}}\end{array}}\right\}$$

$\:\rho\:$- density [kg/m³];

$\:{\rho\:}_{o}$- reference density, here: 1000 [kg/m³];

$$\:\varvec{v\:}$$- velocity vector [m/s];

$\:e$- specific energy [J/kg];

$$\:p$$- pressure [Pa];

$$\:\varvec{I}$$- unit tensor [-];

$\:k$- thermal conductivity coefficient [W/mK];

$\:\varvec{\tau\:}$- total irreversible momentum flux [Pa];

$$\:T$$- temperature [K]

 $\:\varvec{g}$- gravity acceleration vector [m/s^2^];

$\:{\dot{q}}_{\text{A}}$- source of the useful energy as a result of laser irradiation [W/m³].

To enhance the readability of equations and streamline notation, the following simplifications were introduced^30,31,59^:

Source of energy in front of human skin:

$$\:{\dot{q}}_{{\text{P}\text{B}\text{S}}_{\text{I}}}=\frac{{I}_{{\text{abs PBS}}_{I}}}{{D}_{{PBS}_{I}}}=$$

$$= \left( {I_{{pd}} \cdot G \cdot H \cdot \left( {1 - \exp \left( { - D_{{PBS}} \cdot \mu _{{{\text{ a PBS}}}} } \right)} \right)} \right) \cdot D_{{{\text{PBS}}_{{\text{I}}} }} ^{{ - 1}} +$$

2 $$\:-\left({I}_{pd}\cdot G\cdot H\cdot \text{exp}\left(-{D}_{{PBS}_{I}}\cdot \left({\mu\:}_{\text{a PBS}}+{\mu\:}_{\text{s PBS}}\right)\right)\right)\cdot {{D}_{{\text{P}\text{B}\text{S}}_{\text{I}}}}^{-1}$$

Source of energy of epidermis:

$$\:{\dot{q}}_{\text{e}\text{p}}=\frac{{I}_{\text{abs ep}}}{{D}_{\text{e}\text{p}}}=$$

$$\:=\left({I}_{pd}\cdot G\cdot I\cdot \text{exp}\left(-{D}_{{\text{P}\text{B}\text{S}}_{\text{I}}}\cdot \left({\mu\:}_{\text{a PBS}}+{\mu\:}_{\text{s PBS}}\right)\right)\cdot J\cdot \left(1-\text{exp}\left(-{D}_{\text{e}\text{p}}\cdot {\mu\:}_{\text{a ep}}\right)\right)\right)\cdot {{D}_{\text{e}\text{p}}}^{-1}+$$

3 $$\:-\left({I}_{pd}\cdot G\cdot I\cdot \text{exp}\left(-{D}_{{\text{P}\text{B}\text{S}}_{\text{I}}}\cdot \left({\mu\:}_{\text{a PBS}}+{\mu\:}_{\text{s PBS}}\right)\right)\cdot J\cdot \text{exp}\left(-{D}_{\text{e}\text{p}}\cdot \left({\mu\:}_{\text{a ep}}+{\mu\:}_{\text{s ep}}\right)\right)\right)\cdot {{D}_{\text{e}\text{p}}}^{-1}$$

Source of energy of dermis:

$$\:{\dot{q}}_{\text{d}\text{e}\text{r}}=\frac{{I}_{\text{a}\text{b}\text{s}\ \text{d}\text{e}\text{r}}}{{D}_{\text{d}\text{e}\text{r}}}=$$

$$= \left( \begin{gathered} I_{{pd}} \cdot G \cdot I \cdot \exp \left( { - D_{{{\text{PBS}}_{{\text{I}}} }} \cdot \left( {\mu _{{{\text{a}}\ {\text{PBS}}}} + \mu _{{{\text{s}}\ {\text{PBS}}}} } \right)} \right) \cdot J \cdot \exp \left( { - D_{{{\text{ep}}}} \cdot \left( {\mu _{{{\text{a}}\ {\text{ep}}}} + \mu _{{{\text{s}}\ {\text{ep}}}} } \right)} \right) \cdot K \cdot \left( {1 - \exp \left( { - D_{{{\text{der}}}} \cdot \mu _{{{\text{a}}\ {\text{der}}}} }\right)}\right) \hfill \\\end{gathered}\right)\cdot D_{{{\text{der}}}} ^{{ - 1}} +$$

4 $$- \left( \begin{gathered} I_{{pd}} \cdot G \cdot I \cdot \exp \left( { - D_{{{\text{PBS}}_{{\text{I}}} }} \cdot \left( {\mu _{{{\text{a PBS}}}} + \mu _{{{\text{s PBS}}}} } \right)} \right) \cdot J \cdot \exp \left( { - D_{{{\text{ep}}}} \cdot \left( {\mu _{{{\text{a ep}}}} + \mu _{{{\text{s ep}}}} } \right)} \right) \cdot K \cdot \exp \left( { - D_{{{\text{der}}}} \cdot \left( {\mu _{{{\text{a der}}}} + \mu _{{{\text{s der}}}} } \right)} \right) \hfill \\ \end{gathered} \right) \cdot D_{{{\text{der}}}} ^{{ - 1}}$$

Source of energy behind human skin:

$$\:{\dot{q}}_{{\text{P}\text{B}\text{S}}_{\text{I}\text{I}}}=\frac{{I}_{{\text{abs PBS}}_{\text{I}\text{I}}}}{{D}_{{\text{P}\text{B}\text{S}}_{\text{I}\text{I}}}}=$$

$$\begin{aligned} &= \bigg( I_{{pd}} \cdot G \cdot I \cdot \exp \left( { - D_{{PBS_{I} }} \cdot \left( {\mu _{{{\text{a}}\ {\text{PBS}} }} + \mu _{{{\text{s}}\ {\text{PBS}} }} } \right)} \right) \cdot J \cdot \exp \left( { - D_{{ep}} \cdot \left( {\mu _{{{\text{a}}\ {\text{ep}} }} + \mu _{{{\text{s}}\ {\text{ep}} }} } \right)} \right)\\&\quad \cdot K\exp \left( { - D_{{der}} \cdot \left( {\mu _{{\text{a der}}} + \mu _{{\text{s der}}} } \right)} \right) \cdot L \cdot \left( {1 - \exp \left( { - D_{{PBS_{{II}} }} \cdot \mu _{{\text{a PBS}}} } \right)} \right)  \bigg) \cdot D_{{PBS_{{II}}}} ^{{ - 1}} +\end{aligned}$$

5 $$\begin{aligned} &- \bigg(I_{{pd}} \cdot G \cdot I \cdot \exp \left( { - D_{{{\text{PBS}}_{{\text{I}}} }} \cdot \left( {\mu _{{{\text{a PBS}}}} + \mu _{{{\text{s PBS}}}} } \right)} \right) \cdot J \cdot \exp \left( { - D_{{{\text{ep}}}} \cdot \left( {\mu _{{{\text{a ep}}}} + \mu _{{{\text{s ep}}}} } \right)} \right) \\&\quad \cdot K\exp \left( { - D_{{{\text{der}}}} \cdot \left( {\mu _{{{\text{a der}}}} + \mu _{{{\text{s der}}}} } \right)} \right) \cdot L\cdot \exp \left( { - D_{{{\text{PBS}}_{{{\text{II}}}} }} \cdot \left( {\mu _{{{\text{a PBS}}}} + \mu _{{{\text{s PBS}}}} } \right)} \right)  \bigg) \cdot D_{{{\text{PBS}}_{{{\text{II}}}} }} ^{{ - 1}}\end{aligned}$$

Furthermore, power density ($\:{I}_{pd}$ ) with the Gaussian beam profile:

6 $$\:{I}_{pd}=\frac{{P}_{o}\cdot \text{exp}\left(-2\cdot {\left(\frac{r}{\mathcal{B}}\right)}^{2}\right)}{\pi\:\cdot {\mathcal{B}}^{2}}\cdot \frac{\underset{0}{\overset{\mathcal{B}}{\int\:}}\left(\underset{0}{\overset{2\pi\:}{\int\:}}\left(r\cdot \text{exp}\left(-2\cdot {\left(\frac{r}{B}\right)}^{2}\right)\right)d\varphi\:\right)dr}{\underset{0}{\overset{\mathcal{B}}{\int\:}}\left(\underset{0}{\overset{2\pi\:}{\int\:}}\left(r\right)d\varphi\:\right)dr}$$

where:

$\:{\dot{q}}_{{\text{P}\text{B}\text{S}}_{\text{I}}}$ – heat source of PBS right behind the cuvette’s glass [W/m^3^];

$\:{\dot{q}}_{\text{e}\text{p}}$ – heat source of epidermis [W/m^3^];

$\:{\dot{q}}_{\text{d}\text{e}\text{r}}$ – heat source of dermis [W/m^3^];

$\:{\dot{q}}_{{\text{P}\text{B}\text{S}}_{\text{I}\text{I}}}$ – heat source of PBS right behind the dermis layer [W/m^3^];

$\:{I}_{\text{abs A}}$ – absorbed part of irradiation of the A part [W/m^2^];

$\:{D}_{A}$ – optical path of the A material [m];

$\:{\mu\:}_{\text{a A}}$ – absorption coefficient of the A material [1/m];

$\:{\mu\:}_{\text{s A}}$ – scattering coefficient of the A material [1/m];

$$\:r$$,$$\:\varphi\:$$ – cylindrical coordinates;

$\:\mathcal{B}$ – beam size, here: 0.004 [m];

$\:{PBS}_{I}$ – phosphate buffered saline right behind the cuvette’s glass;

$\:pd$ – power density. The following coefficients appearing in equations (2-5) should be highlighted:

7 $$\:G=1-{R}_{\text{c}\text{u}\text{v}/\text{a}\text{i}\text{r}}$$

8 $$\:H=1-{R}_{\text{g}\text{l}/\text{P}\text{B}\text{S}}$$

9 $$\:I=1-{R}_{\text{c}\text{u}\text{v}/\text{P}\text{B}\text{S}}$$

10 $$\:J=1-{R}_{\text{P}\text{B}\text{S}/\text{e}\text{p}}$$

11 $$\:K=1-{R}_{\text{e}\text{p}/\text{d}\text{e}\text{r}}$$

12 $$\:L=1-{R}_{\text{d}\text{e}\text{r}/\text{P}\text{B}\text{S}}$$

13 $$\:{R}_{A/B}=\frac{{\left(\stackrel{-}{{n}_{A}}-\stackrel{-}{{n}_{B}}\right)}^{2}+{\left(\stackrel{\prime}{{n}_{A}}-\stackrel{\prime}{{n}_{B}}\right)}^{2}}{{\left(\stackrel{-}{{n}_{A}}+\stackrel{-}{{n}_{B}}\right)}^{2}+{\left(\stackrel{\prime}{{n}_{A}}+\stackrel{\prime}{{n}_{B}}\right)}^{2}}$$

where:

$\:{R}_{A/B}$ – Fresnel’s reflection coefficient at the boundary between A and B materials;

$\:\stackrel{-}{{n}_{A}}$ – real part of A’s refractive index;

$\:\stackrel{-}{{n}_{B}}$ – real part of B’s refractive index;

$\:\stackrel{\prime}{{n}_{A}}$ – imaginary part of A’s refractive index;

$\:\stackrel{\prime}{{n}_{B}}$ – imaginary part of B’s refractive index.

The heat generation of nanostructures is modelled differently due to the size-dependent properties and the interaction with the electrical field. Theoretical frameworks were introduced by Mie, who determined optical cross-sections of gold nanospheres^59,60^ or Bohren, who considered energy conversion based on the electrical field distribution^60,61^. Following these assumptions, heat generation and plasmon resonance in laser-irradiated nanoparticles can be determined by the electrical effective polarizability and the absorption and scattering coefficients, yielding:

14 $$\:{\mu\:}_{\text{a Au}}={\xi\:}_{Au}\cdot {\mathcal{C}}_{a}={\xi\:}_{Au}\cdot \left({\eta\:}_{PT}\cdot 4\pi\:\cdot \left(\frac{2\pi\:}{\lambda\:}\right)\cdot \left(\stackrel{\prime}{{\alpha\:}_{\text{e}\text{f}\text{f}}}\right)\right)$$

15 $$\:{\mu\:}_{\text{s Au}}={\xi\:}_{Au} \cdot {\mathcal{C}}_{s}={\xi\:}_{Au}\cdot \left(\frac{8\pi\:}{3}\cdot {\left(\frac{2\pi\:}{\lambda\:}\right)}^{4}\cdot \left({\left(\stackrel{\prime}{{\alpha\:}_{\text{e}\text{f}\text{f}}}\right)}^{2}+{\left(\stackrel{-}{{\alpha\:}_{\text{e}\text{f}\text{f}}}\right)}^{2}\right)\right)$$

16 $$\:{\eta\:}_{PT}=1-\left(\frac{2}{3}\cdot {\left(\frac{2\pi\:}{\lambda\:}\right)}^{3}\cdot \frac{{\left(\stackrel{\prime}{{\alpha\:}_{\text{e}\text{f}\text{f}}}\right)}^{2}+{\left(\stackrel{-}{{\alpha\:}_{\text{e}\text{f}\text{f}}}\right)}^{2}}{\mathbbm{i} \mathbbm{m}\left(\stackrel{\prime}{{\alpha\:}_{\text{e}\text{f}\text{f}}}\right)}\right)$$

where:

$\:{\xi\:}_{Au}$ – particle number concentration;

$\:{\mathcal{C}}_{a}$ – absorption cross section of the nanoparticles;

$\:{\mathcal{C}}_{s}$ – scattering cross section of the nanoparticles;

$\:\stackrel{\prime}{{\alpha\:}_{\text{e}\text{f}\text{f}}}$ – imaginary part of electrical polarizability of the nanoparticles;

$\:\stackrel{-}{{\alpha\:}_{\text{e}\text{f}\text{f}}}$ – real part of electrical polarizability of the nanoparticles;

$$\:{\lambda}$$ – laser wavelength.

As may be noticed, the approach advantages to calculate explicitly one, effective parameter, which is the electrical polarizability. The parameter can follow different models, such as in^27,62,63^. In this paper, detailed methodology associated with localized electrical field contribution was taken from the authors’ previous works^10,39,50,59,60^ in which detailed model calibration and verification on various experiments were performed.

**Fig. 7:**
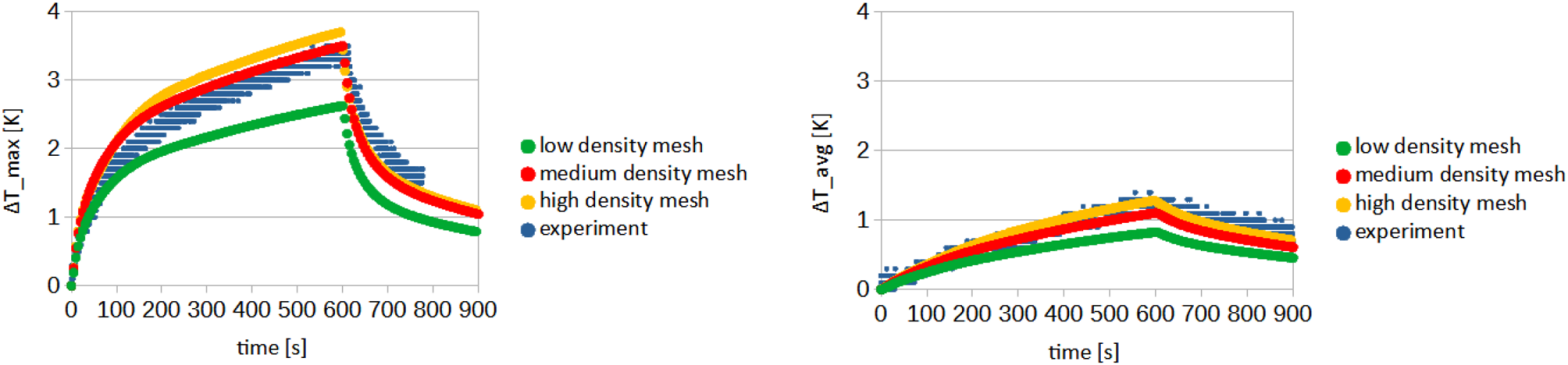
Comparison of temperature increase curves obtained during experiment and as a result of performed simulations.

A mesh quality test was included, and the results (Fig. 7; Table 3) indicated that a medium-density mesh is sufficient for accurately estimating the temperature distribution within the tissue. This mesh configuration provides satisfactory results while maintaining a balance between computational efficiency and accuracy. Although the high-density mesh yields slightly more precise results, the associated computational time increases significantly, making it less practical for the current study.

Basic mesh quality parameters, including average skewness, element quality, orthogonality, and aspect ratio, were evaluated and compared. The obtained values are summarized in (Table 3). It is also worth noting that the presented model not only enables the replication of experimentally observed processes but also, in the long term, facilitates the optimization of both nanoparticles and the analyzed procedures^51,61^.

**Table 3:** Results of mesh quality test.

	Mesh density		
	Low	Medium	High
Element size	0.3 mm	0.2 mm	0.1 mm
Number of nodes	286,889	1,480,656	5,112,377
Number of elements	459,539	2,158,913	8,288,187
Average skewness	0.213	0.194	0.187
Average element quality	0.726	0.711	0.698
Average aspect ratio	2.425	2.373	2.507
Average orthogonal quality	0.839	0.865	0.864

The boundary conditions for the cuvette walls were defined as ‘mixed boundary conditions’, incorporating both convection and radiation effects to enhance the accuracy of thermal analysis. The heat transfer coefficient was set to 5 W/(m²K)^50^. The external emissivity was assigned a value of 0.95, which was calibrated for the quartz glass. The SIMPLE algorithm was selected for the calculations due to its balance between computational efficiency and accuracy, eliminating the necessity for more complex and time-intensive methods.

For pressure calculations, the PRESTO! scheme was employed, as it is specifically designed for processes involving natural convection. Meanwhile, the “second-order upwind” scheme was applied for momentum, turbulence kinetic energy, and dissipation rate to improve numerical stability and accuracy. The total number of time steps was set to 9,000, with a time increment of 0.1 s. The time step size was determined using the following equation:

17 $$\:{t}_{s}=\frac{{\alpha\:}^{2}}{a}=\frac{{\alpha\:}^{2}}{\frac{k}{{c}_{p}\cdot \:\rho\:}}=\frac{{\alpha\:}^{2}\cdot \:{c}_{p}\cdot \:\rho\:}{k}$$

$\:{t}_{s}$- time step size (increment) [s];

$\:\alpha\:$- smallest element dimension in the model [m];

$\:a$- largest thermal diffusivity [m²/s];

$\:{c}_{p}$- specific heat [J/(kgK)];

$\:\rho\:$- density [kg/m³];

$k\:$- thermal conductivity [W/(mK)].

### Histological and LDH analysis

In the article, it was also relevant to determine the effect of individual wavelengths and lasers on irradiated skin tissue. This part aimed to determine the wavelengths at which it is justified to inject gold nanoparticles into the skin and then irradiate them. Material from skin tissue was fixed using formalin solution (Sigma – Aldrich cat. nr 1004968350). After 7 days, the fixed biological material was rinsed under running water. The next stage was the dehydration of the fixed tissue fragment by passing it through a series of alcohols of increasing concentration. After completing this stage, the preparations were passed through intermediate fluids - at this point, the biological material was incubated in xylene (POCH, cat. nr 520860119). Next, the preparation was incubated in a paraffin solution (Merck, cat nr 1116092504) at 58 degrees Celsius and then finally embedded in paraffin. Once the paraffin-embedded tissue had set, it was possible to cut the material into ultra-thin Sect. (5 μm) using a microtome Leica SM 2000R. The sections with the material were placed on glass slides (Menzel – Gläser, cat. nr J2800AMNZ) and allowed to dry. The slides prepared in this way were stained using Masson’s method with Goldner’s modification. Photographic documentation of the prepared microscopic slides was performed using an inverted microscope Leica model DM IL LED.

Cytotoxicity was tested on skin samples by analyzing the activity of lactate dehydrogenase (LDH, Roche, cat. nr.: 11644793001). Skin samples were previously exposed to 10 min of irradiation with the following laser beams wavelength: 980 nm, 808 nm, 640 nm, 532 nm, 465 nm. Skin samples not exposed to laser irradiation served as negative controls, while Triton X-100 detergent (Merck, cat. nr. X100-500ML) was used as positive control - max. 100% cytotoxicity. After irradiation, skin samples were placed on 24-well plates and incubated under standard conditions. After 24 h of incubation, supernatants were collected for LDH analysis, which was performed according to the manufacturer’s instructions. Samples were read using a Synergy LX spectrophotometer from Biotek, at a wavelength of 450 nm. Results were presented as percentage of cytotoxicity using the formula:

18 $$\:\text{\%}\:\text{c}\text{y}\text{t}\text{o}\text{t}\text{o}\text{x}\text{i}\text{c}\text{i}\text{t}\text{y}=\frac{\text{A}-\text{K}}{{\text{K}}_{\text{T}}-\text{K}}\cdot \:100\text{\%}$$

where:

$$\:\text{A}-\:$$tested sample;

$\:\text{K}$ – control (minimal cytotoxicity);

$\:{\text{K}}_{\text{T}}$ – positive control (maximum cytotoxicity).

## Experiment results

**Fig. 8:**
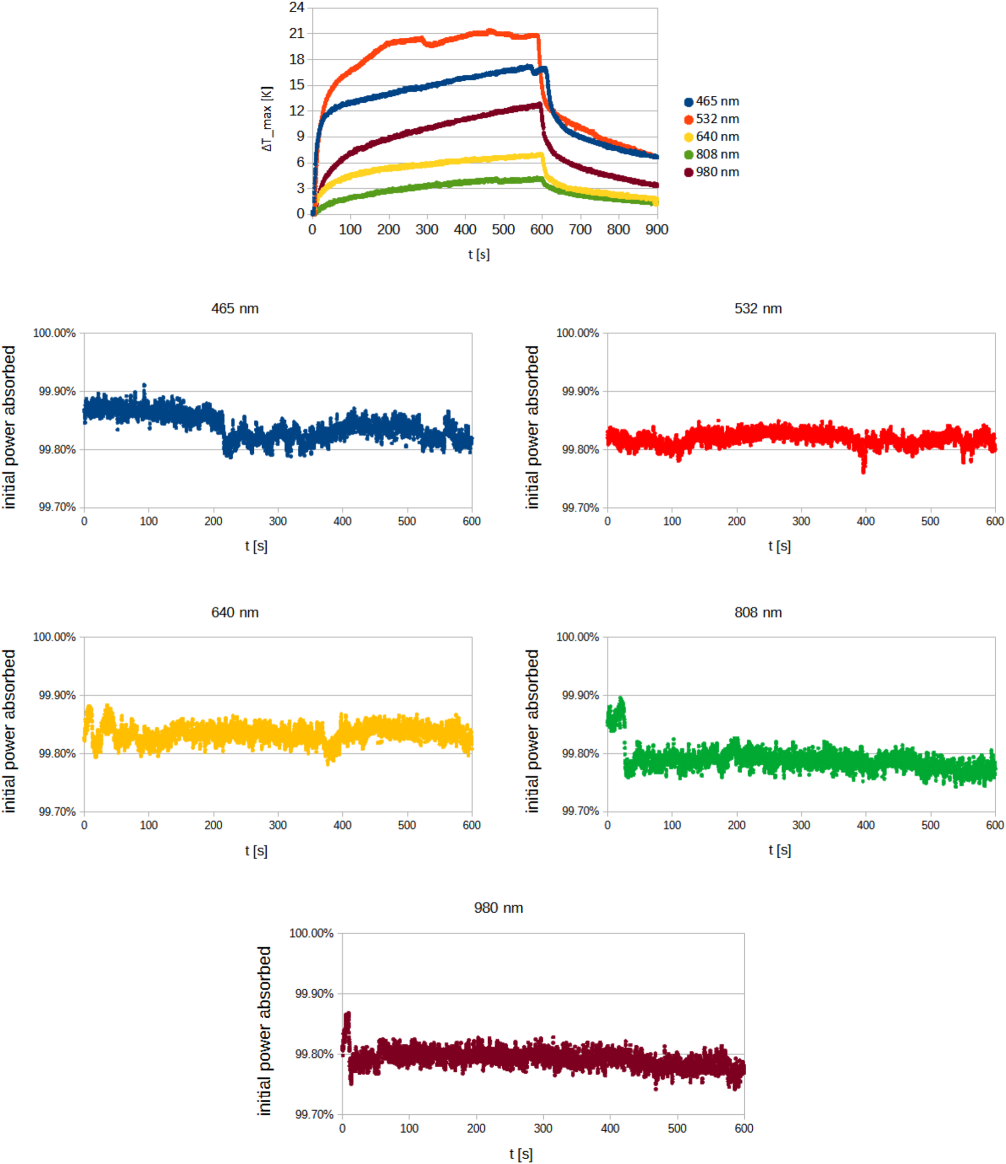
Temperature increase after 10 min of irradiation and power absorbed by the system – comparison of five different laser wavelengths.

**Fig. 9:**
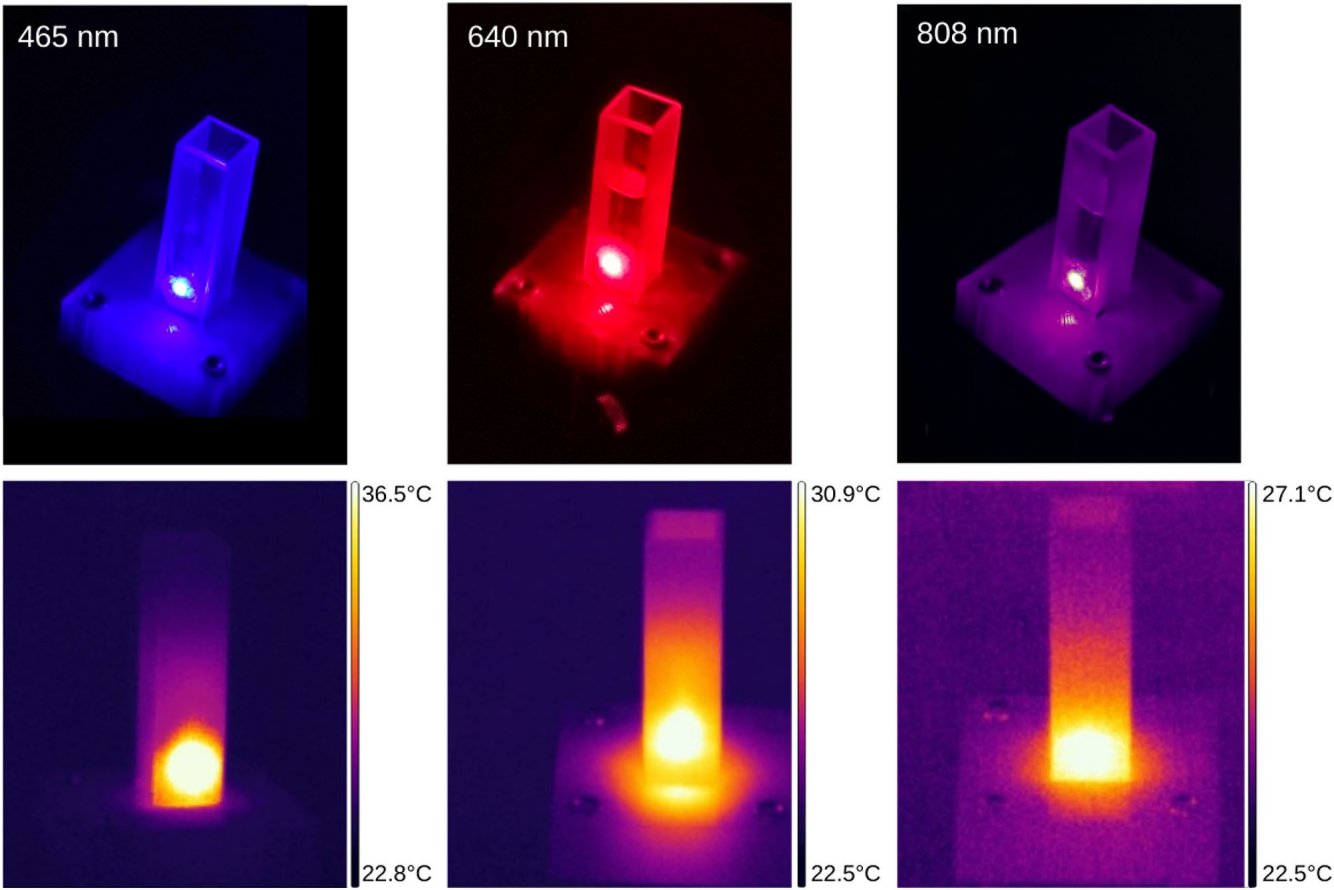
Exemplary photos from the experiment and corresponding images from thermal camera after 5 min of irradiation.

As previously stated, the experimental procedure was divided into two separate stages. In the first stage, the effect of 10-minute laser irradiation on human skin samples was examined. The investigation involved five different laser wavelengths: 465 nm, 532 nm, 640 nm, 808 nm, and 980 nm. The temperature increase was measured on the front wall of the glass cuvette using a thermal camera. Simultaneously, the laser power absorbed by the system was quantified using a laser power meter. For each wavelength, six samples were analyzed, and the corresponding results are presented in (Table 4). Figures 8 and 9 present exemplary results obtained from the laser power meter, temperature increase curves, and photographs from the experiment, sequentially. Additionally, corresponding thermal camera images are included to illustrate the temperature distribution during the irradiation process (cf. Fig. 9 bottom).

As may be realized, data from the laser power meter in Table 4 suggests that laser beams were incapable of penetrating the considered system. The small percentage, which takes no more than 0.3%, can be either the transmitted or the externally scattered part of irradiation. Bearing in mind that quartz glass and PBS are optically transparent in the visible range, virtually all electromagnetic radiation is either absorbed or scattered by human skin.

Based on the data in Fig. 8, it can be noticed that each wavelength of the laser produces a markedly different effect in the irradiation process. The differences in the values of temperatures obtained can be explained on the basis of the differences in values of the absorption and scattering coefficients in Table 2, which directly affect the efficiency of conversion of electromagnetic energy into heat. However, the shape of temperature curves indicates the heating of the PBS solution itself, whose properties are similar to water. It can be noted that for the red (640 nm) and infrared (808 nm) laser beams, PBS does not absorb radiation, which was also confirmed in^57^.

In addition, 465 and 532 nm wavelengths are significantly different from the others, where the irregular and jagged shape of the curve in the final irradiation process suggests that structural changes are taking place in theskin, which indicates degradation of healthy skin cells and rules them out for use in photothermal tumor therapy with nanoparticles.

**Table 4:** Temperature increase after 10 min of irradiation and average power absorbed by the system – comparison of five different laser wavelengths.

Laser wavelength [nm]	Maximum temperature, T_max [°C]	ΔT_max [°C]	Average ΔT_max [°C]	Laser power [W]	Power absorbed by the system (percentage of initial power absorbed) [W]
465	32.1	9.2	14.3	0.8	0.7987 (99.841%)
	49.2	24.1			
	43.6	16.7			
	33.3	7.5			
	42	17.3			
	34.7	11.1			
532	34.8	12.2	19.6	1.1	1.0980 (99.819%)
	50.9	27.5			
	35.4	12.5			
	37.8	14.4			
	54.4	29.3			
	45.7	21.4			
640	28.5	3.8	8.7	0.8	0.7987 (99.834%)
	37.3	12.6			
	30.9	6.5			
	31.5	7.0			
	40.7	16.5			
	29.1	5.6			
808	27.4	4.2	3.9	0.8	0.7983 (99.789%)
	27.9	4.3			
	27.1	3.0			
	26.8	3.5			
	26.6	2.0			
	29.2	6.4			
980	36.5	12.1	11.0	0.8	0.7983 (99.792%)
	38.5	12.9			
	34.3	10.1			
	34.5	10.0			
	36.5	11.3			
	34.3	9.8			

**Fig. 10:**
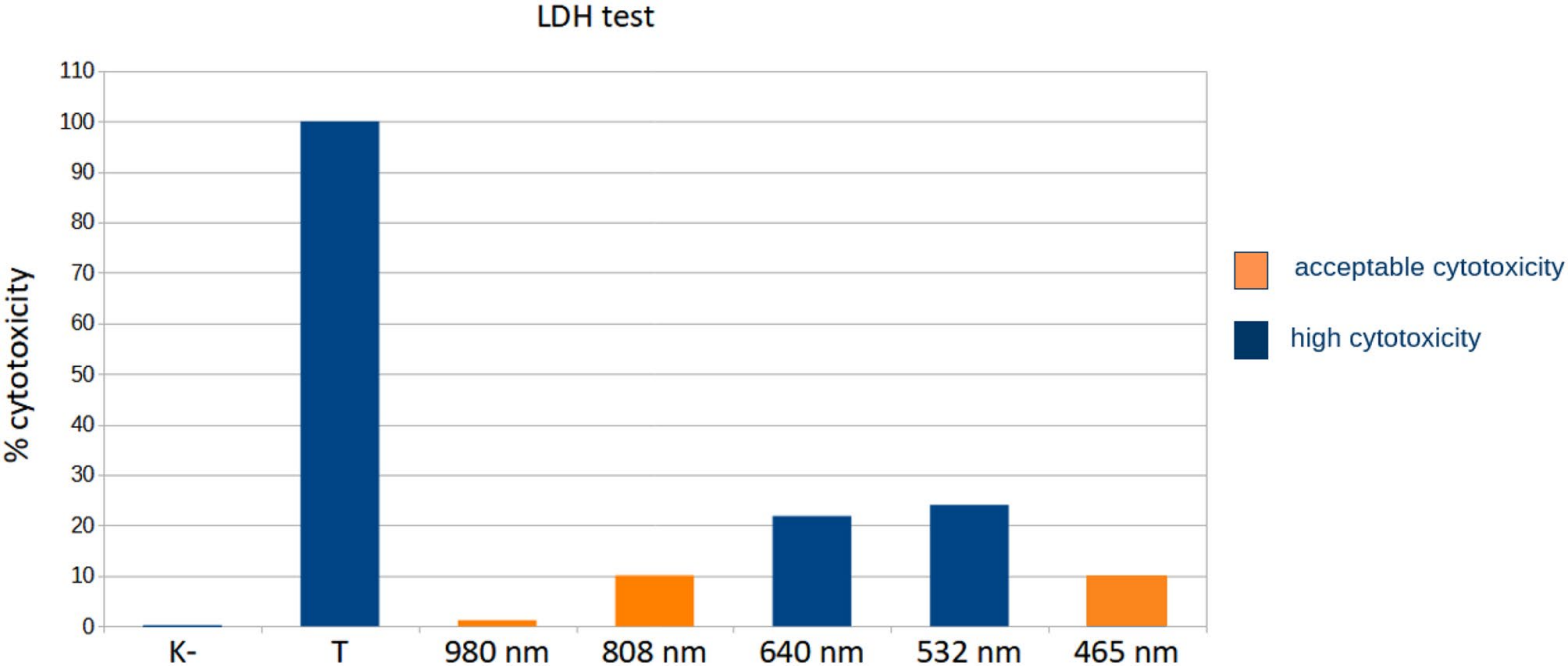
LDH test results of 10-min laser exposure of five different laser wavelengths.

Moreover, the cytotoxicity tests were conduct to evaluate the potential changes in the skin tissue after laser irradiation. The obtained results for each used laser wavelength are visible in (Fig. 10). As may be noticed, the 532 nm and 640 nm laser irradiation exhibited high cytotoxicity effect, which would contribute to a damage and destruction of the epidermis layer. Based on these findings, the other wavelengths are considered as more suitable for exposure in terms of nanoparticles injection and tumors ablation.

**Fig. 11:**
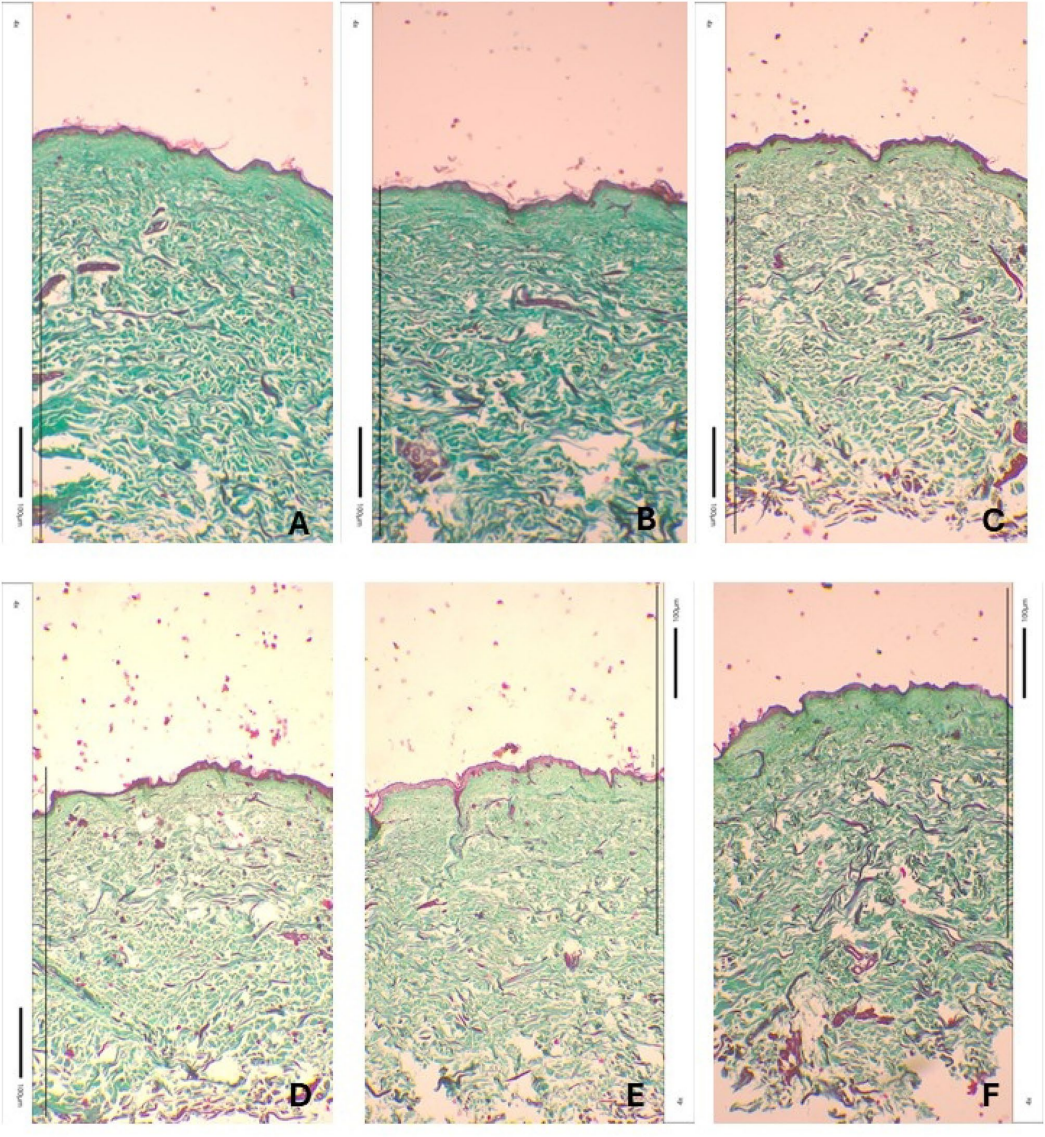

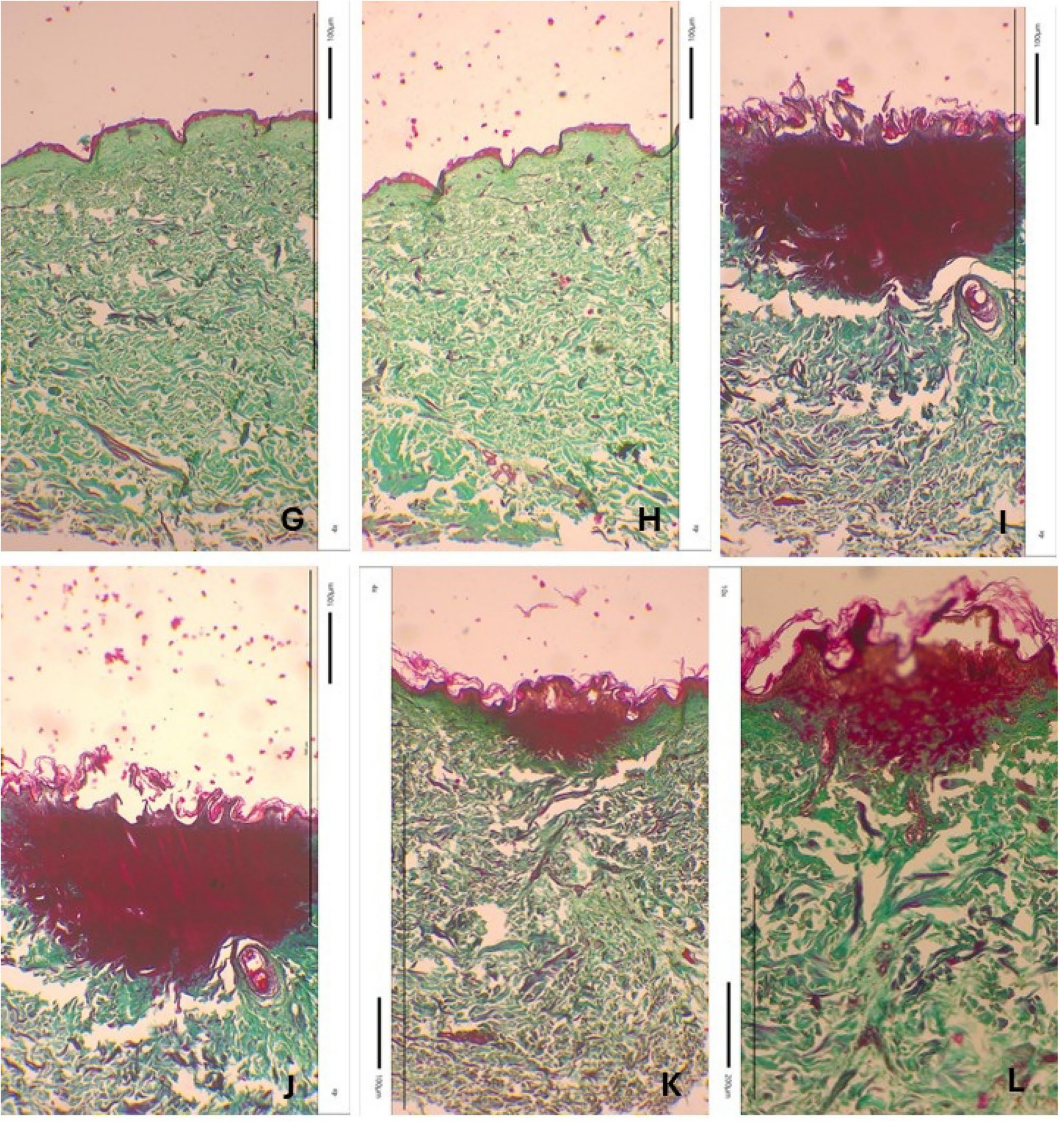
Skin samples stained using Masson’s method with Goldner’s modification. control – a view of a skin fragment not exposed to the laser (**A**,**B**) and a view of a skin fragment exposed to individual laser wavelengths: 980 nm (**C**,**D**), 808 nm (**E**,**F**), 640 nm (**G**,**H**), 532 nm (**I**,**J**), 465 nm (**K**,**L**). Photos taken with an inverted light microscope Leica model DM IL LED.

To analyze and visualize the impact of laser exposure on skin structure, the histological examination were used. The results in Fig. 11 presents skin fragments, - A, B not exposed to laser, and skin fragments exposed to laser: 980 nm – Fig. 11C and D, 808 nm - Fig. 11E and F, 640 nm – Fig. 11G and H, 532 nm – Fig. 11I and J, 465 nm – Fig. 11K, L. The staining results show cell nuclei as brownish black, fibrin - red, connective tissue - green^64,65^. No changes in the tissue caused by laser action were observed in samples exposed to 980 nm, 808 nm and 640 nm. However, the 532 nm and 465 nm laser beams caused the destruction of the first layer of the skin - the epidermis. The image of photos from skin staining using the Masson method with Goldner’s modification clearly indicated that the 532 nm and 465 nm laser beams caused epidermal necrosis, therefore these laser beams were rejected for further experiments. Furthermore, cytotoxicity tests also exclude the 640 nm wavelength.

**Fig. 12:**
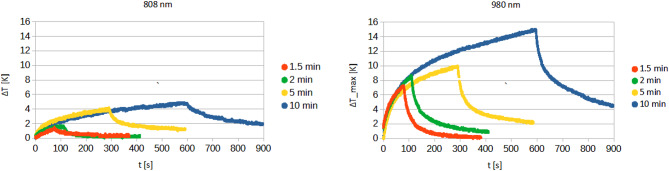
Comparison of temperature increase for different irradiation time (1.5, 2, 5, 10 min) for 808 and 980 nm lasers.

**Fig. 13:**
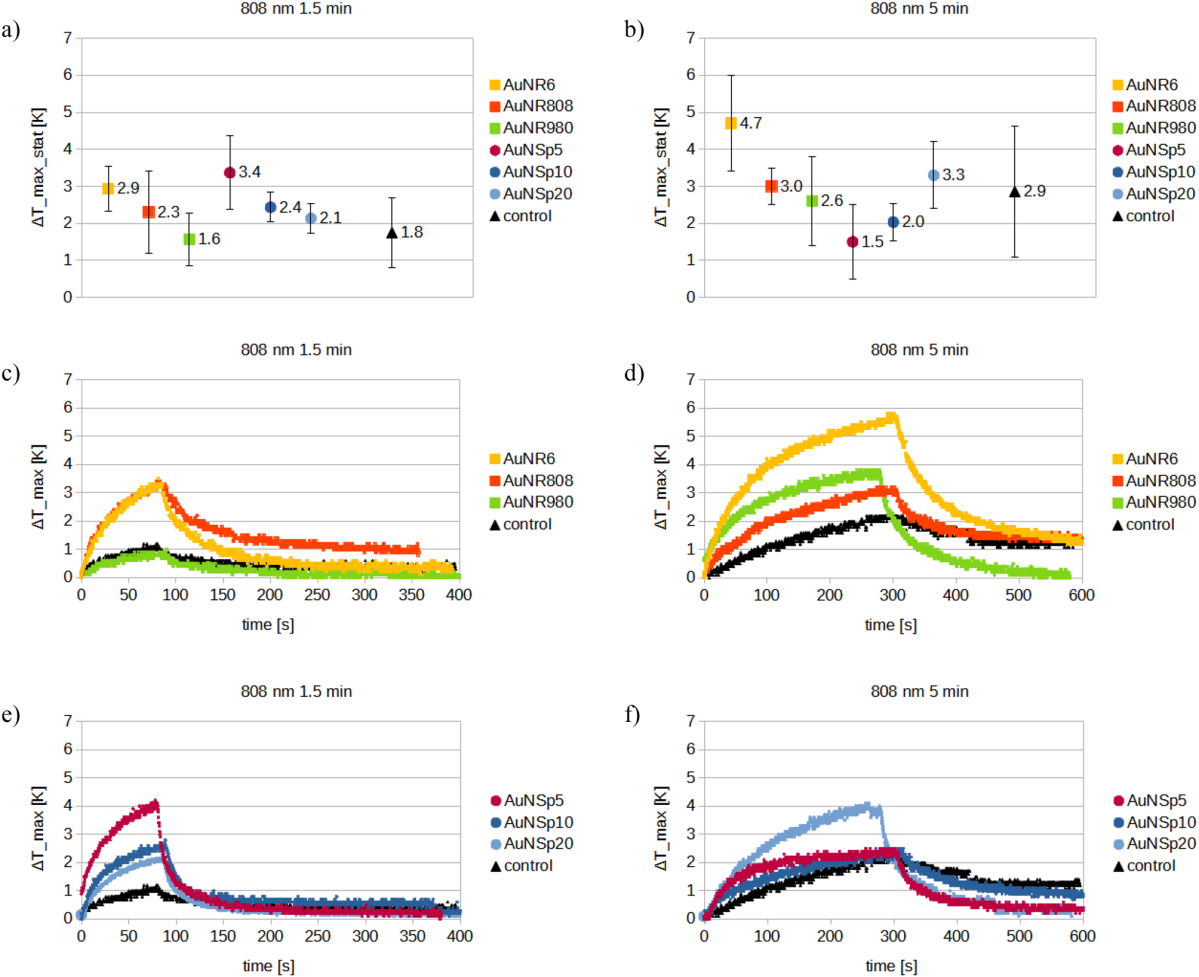
808 nm laser, comparison of: average temperature increase values for (**a**) 1.5 min of irradiation, (**b**) 5 min of irradiation; temperature increase curves after (**c**,**d**) nanorods injection, (**e**,**f**) nanospheres injection. The “ΔT_max_stat” takes the statistically averaged value of maximum temperature increase.

**Fig. 14:**
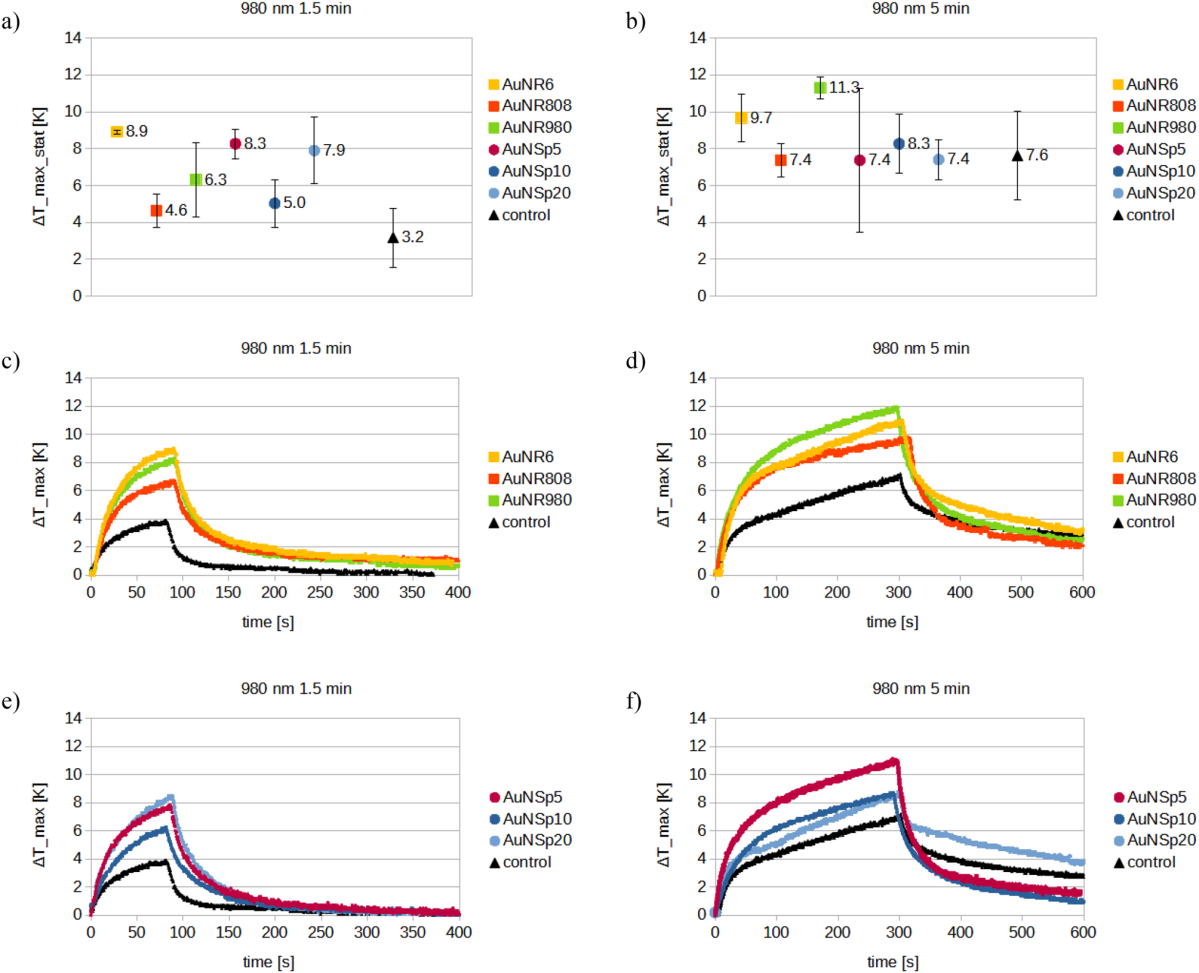
980 nm laser, comparison of: statistically averaged temperature increase values (ΔT_max_stat) for (**a**) 1.5 min of irradiation, (**b**) 5 min of irradiation; temperature increase curves after (**c**,**d**) nanorods injection, (**e**,**f**) nanospheres injection (error bars were stablished as a maximum data uncertainty value).

Following data in literature^52–54^, the near-infrared region is studied also due the minimal optical density of human skin, which contributes to reduce the risk of metastases and recurrences after surgical operations of health tissues. Therefore, research was focused for the next stage of the experiment on two laser wavelengths: 808 and 980 nm. Figure 12 demonstrates the temperature profiles due to laser radiation at 0.8 W initial power for 808 and 980 nm wavelengths after 1.5, 2.0, 5.0 and 10.0 min. Regarding the irradiation duration, exposure times of 1.5 min and 5 min were chosen to further research. To assess the temperature increase in the absence of nanoparticles, ten control samples were used for each wavelength, where only human skin samples were irradiated. In the subsequent step, 15 µl of the selected nanoparticles solution (Table 1) was injected into the samples, followed by irradiation for the predetermined durations. Three samples were used for each nanoparticle solution. All obtained results, including exemplary temperature increase curves, are presented in (Tables 5, 6 and 7; Figs. 13 and 14).

**Table 5:** Results of temperature increase after irradiation with λ=808 nm laser for control samples and samples with injected nanoparticles.

Temperature increase – all results for 808 nm laser				
Irradiance time [min]	Used nanoparticles	Maximum temperature, T_max [°C]	ΔT_max [°C]	Average ΔT_max [°C]
1.5	-	24.0	1.5	1.8 ± 1.0
1.5	-	25.7	1.8	
1.5	-	25.2	2.0	
1.5	-	24.4	2.2	
1.5	-	23.3	1.2	
1.5	-	24.5	1.7	
1.5	-	23.8	0.8	
1.5	-	24.2	1.1	
1.5	-	26.2	2.6	
1.5	-	26.3	2.6	
1.5	AuNR6	27.6	3.5	2.9 ± 0.6
1.5	AuNR6	25.7	2.3	
1.5	AuNR6	25.7	3.0	
1.5	AuNR808	26.6	3.4	2.3 ± 1.1
1.5	AuNR808	22.4	1.4	
1.5	AuNR808	22.9	2.1	
1.5	AuNR980	21.3	1.0	1.6 ± 0.7
1.5	AuNR980	23.0	1.4	
1.5	AuNR980	23.1	2.3	
1.5	AuNSp5	25.4	3.5	3.4 ± 1.0
1.5	AuNSp5	27.7	4.2	
1.5	AuNSp5	21.8	2.4	
1.5	AuNSp10	25.3	2.5	2.4 ± 0.4
1.5	AuNSp10	25.5	2.8	
1.5	AuNSp10	25.8	1.2	
1.5	AuNSp20	24.0	2.0	2.1 ± 0.4
1.5	AuNSp20	24.4	2.5	
1.5	AuNSp20	24.9	1.9	

Temperature increase – all results for 808 nm laser				
Irradiance time [min]	Used nanoparticles	Maximum temperature, T_max [°C]	ΔT_max [°C]	Average ΔT_max [°C]
5	-	27.1	4.2	2.9 ± 1.8
5	-	27.1	3.7	
5	-	25.9	3.2	
5	-	25.1	2.0	
5	-	26.1	2.3	
5	-	23.4	1.1	
5	-	25.4	3.8	
5	-	27.3	3.0	
5	-	28.0	3.8	
5	-	25.1	1.5	
5	AuNR6	28.1	3.4	4.7 ± 1.3
5	AuNR6	30.1	5.8	
5	AuNR6	26.7	4.9	
5	AuNR808	26.3	3.2	3.0 ± 0.5
5	AuNR808	24.1	2.5	
5	AuNR808	24.7	3.3	
5	AuNR980	24.1	1.9	2.6 ± 1.2
5	AuNR980	23.6	2.1	
5	AuNR980	24.6	3.8	
5	AuNSp5	23.9	1.0	1.5 ± 1.0
5	AuNSp5	23.4	1.0	
5	AuNSp5	26.5	2.5	
5	AuNSp10	24.3	1.7	2.0 ± 0.5
5	AuNSp10	23.8	1.9	
5	AuNSp10	28.6	2.5	
5	AuNSp20	25.2	3.5	3.3 ± 0.9
5	AuNSp20	24.4	2.4	
5	AuNSp20	26.4	4.0	

**Table 6:** Results of temperature increase after irradiation with λ=980 nm laser for control samples and samples with injected nanoparticles.

Temperature increase – all results for 980 nm laser				
Irradiance time [min]	Used nanoparticles	Maximum temperature, T_max [°C]	ΔT_max [°C]	Average ΔT_max [°C]
1.5		26.5	3.8	3.2 ± 1.6
1.5	-	29.2	4.8	
1.5	-	26.3	2.6	
1.5	-	26.9	2.8	
1.5	-	24.5	1.6	
1.5	-	25.9	2.6	
1.5	-	25.6	2.0	
1.5	-	26.7	3.9	
1.5	-	25.3	4.1	
1.5	-	24.9	3.6	
1.5	AuNR6	35.0	8.9	8.9 ± 0.1
1.5	AuNR6	33.5	9.0	
1.5	AuNR6	32.4	8.8	
1.5	AuNR808	28.8	4.7	4.6 ± 0.9
1.5	AuNR808	28.8	5.5	
1.5	AuNR808	26.2	3.7	
1.5	AuNR980	30.0	8.3	6.3 ± 2.0
1.5	AuNR980	27.7	4.7	
1.5	AuNR980	24.7	5.9	
1.5	AuNSp5	28.7	7.8	8.3 ± 0.8
1.5	AuNSp5	28.8	7.9	
1.5	AuNSp5	30.0	9.1	
1.5	AuNSp10	30.4	6.3	5.0 ± 1.3
1.5	AuNSp10	25.5	3.7	
1.5	AuNSp10	30.1	5.1	
1.5	AuNSp20	27.5	6.1	7.9 ± 1.8
1.5	AuNSp20	29.1	9.1	
1.5	AuNSp20	27.8	8.5	

Temperature increase – all results for 980 nm laser				
Irradiance time [min]	Used nanoparticles	Maximum temperature, T_max [°C]	ΔT_max [°C]	Average ΔT_max [°C]
5	-	30.0	7.2	7.6 ± 2.4
5	-	31.6	7.5	
5	-	32.2	8.8	
5	-	31.8	8.4	
5	-	29.8	5.6	
5	-	30.3	7.0	
5	-	30.2	8.2	
5	-	29	7.2	
5	-	28.8	6.4	
5	-	32.0	10.0	
5	AuNR6	32.8	8.6	9.7 ± 1.3
5	AuNR6	36.7	11.0	
5	AuNR6	32.7	9.4	
5	AuNR808	30.6	6.5	7.4 ± 3.9
5	AuNR808	30.9	7.8	
5	AuNR808	30.8	7.8	
5	AuNR980	35.0	11.9	11.3 ± 0.6
5	AuNR980	34.8	10.9	
5	AuNR980	32.2	11.1	
5	AuNSp5	34.3	11.3	7.4 ± 3.9
5	AuNSp5	31.7	7.1	
5	AuNSp5	33.7	9.1	
5	AuNSp10	34.5	9.4	8.3 ± 1.6
5	AuNSp10	32.8	8.7	
5	AuNSp10	27.7	6.7	
5	AuNSp20	31.3	8.5	7.4 ± 1.1
5	AuNSp20	29.0	6.6	
5	AuNSp20	27.6	7.1	

As may be noticed, the temperature increases vary differently for all nanoparticles. Among the gold nanorods, AuNR6 offers the greatest results. For 808 nm wavelength, these values ΔT_max were 3.3 and 5.5 °C (Fig. 13), while for 980 nm the temperature increased by 9 and 11 °C for 1.5 and 5 min of irradiation (Fig. 14), respectively. On the other hand, gold nanospheres which do not exhibit any resonance peaks around the incident wavelengths heat surprisingly well, and their values of temperature ΔT_max_stat (cf. Tables 5, 6 and 7) are comparable to those of nanorods.

The absorbance of spherical (citrate-stabilized) gold nanoparticles after skin injection can be attributed to their aggregation into larger clusters, which leads to a red-shifted plasmon resonance and increased extinction values due to coherent coupling^66^. In the skin tissue, these primary particles will be prone to aggregation due to higher ionic strengths and concomitant charge screening and/or replacement of citrate with other competitive ligands that lead to colloidal destabilization. Hence, the nature of the capping agent is key to control/mitigate the plasmon resonance shift within the tissue.

Besides providing limited stability in biological tissue, citrate capping agent has low toxicity, which is confirmed in e.g^67^. Likewise, the PEG capping agent is highly biosafe, but also provides increased colloidal stability to nanoparticles by steric stabilization. There is also the fact that the skin fragments tested came from different patients, which can also mean different pH values, ionic strengths etc. and thus differences in the results. The size of spherical nanoparticles allows them to retain inside skin, they absorb at 808 nm barely, however. It has been demonstrated in literature that human tissues capture small AuNPs with anti-tumor antibodies, and they aggregate catalytically, shifting their absorption into the NIR region^68^. This explains why the utilized nanoparticles exhibit heating at 808 nm, despite the absence of a resonant peak at this wavelength.

**Table 7:** Summary of average temperature increase values for λ=808 and 980 nm lasers.

	ΔT_max_stat [K]			
	λ=808 nm		λ=980 nm	
	1.5 min	5 min	1.5 min	5 min
AuNR6	2.9 ± 0.6	4.7 ± 1.3	8.9 ± 0.1	9.7 ± 1.3
AuNR808	2.3 ± 1.1	3.0 ± 0.5	4.6 ± 0.9	7.4 ± 0.9
AuNR980	1.6 ± 0.7	2.6 ± 1.2	6.3 ± 2.0	11.3 ± 0.6
AuNSp5	3.4 ± 1.0	1.5 ± 1.0	8.3 ± 0.8	7.4 ± 3.9
AuNSp10	2.4 ± 0.4	2.0 ± 0.5	5.0 ± 1.3	8.3 ± 1.6
AuNSp20	2.1 ± 0.4	3.3 ± 0.9	7.9 ± 1.8	7.4 ± 1.1
control	1.8 ± 1.0	2.9 ± 1.8	3.2 ± 1.6	7.6 ± 2.4

## Simulation results

**Fig. 15:**
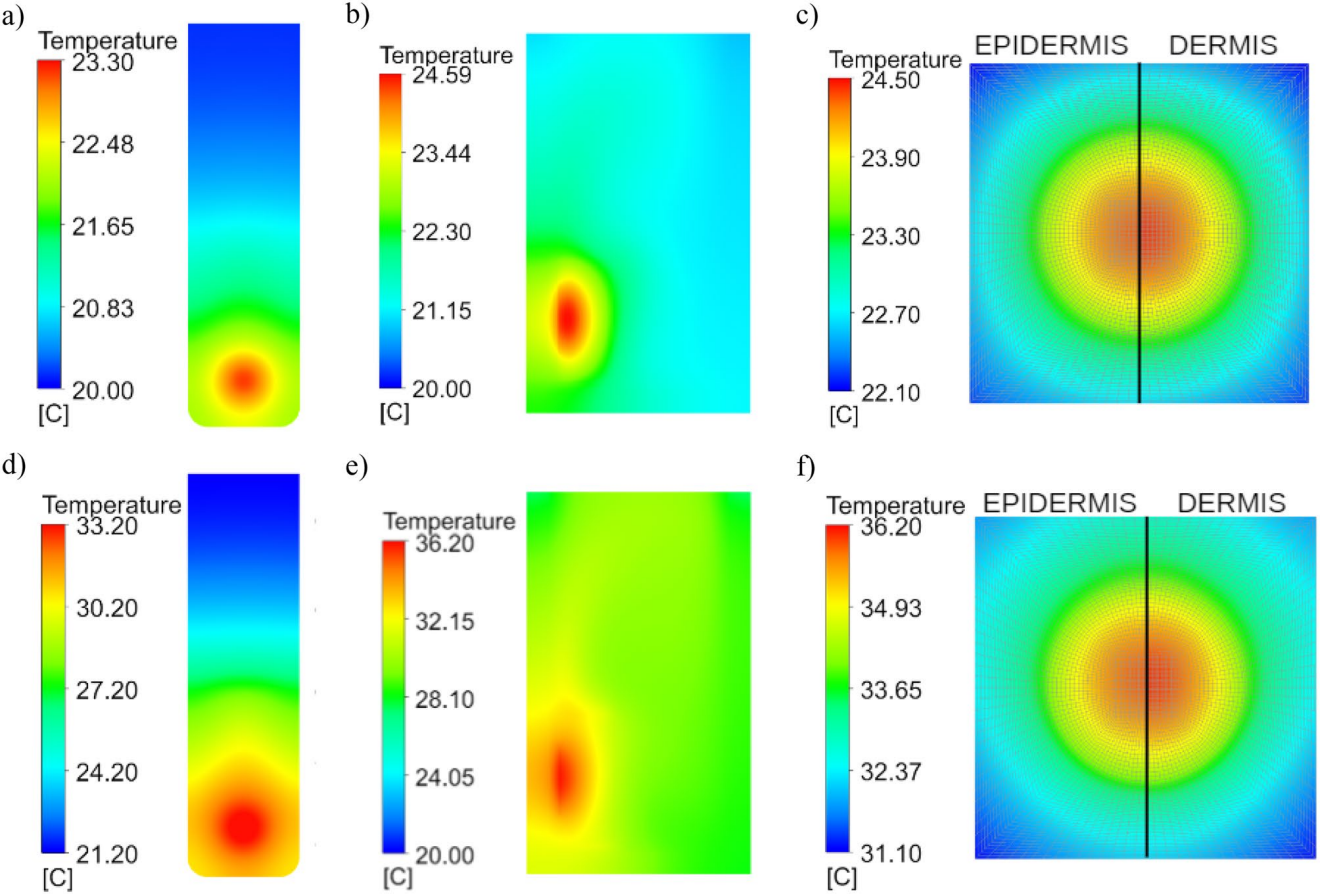
Temperature distribution for the front wall of the cuvette (**a**,**b**), the cross-section of the measurement system (**c**,**d**), and the individual tissue layers (**e**,**f**) – results of simulation: (**a**–**c**) for 808 nm laser, (**d**–**f**) for 980 nm laser.

**Fig. 16:**
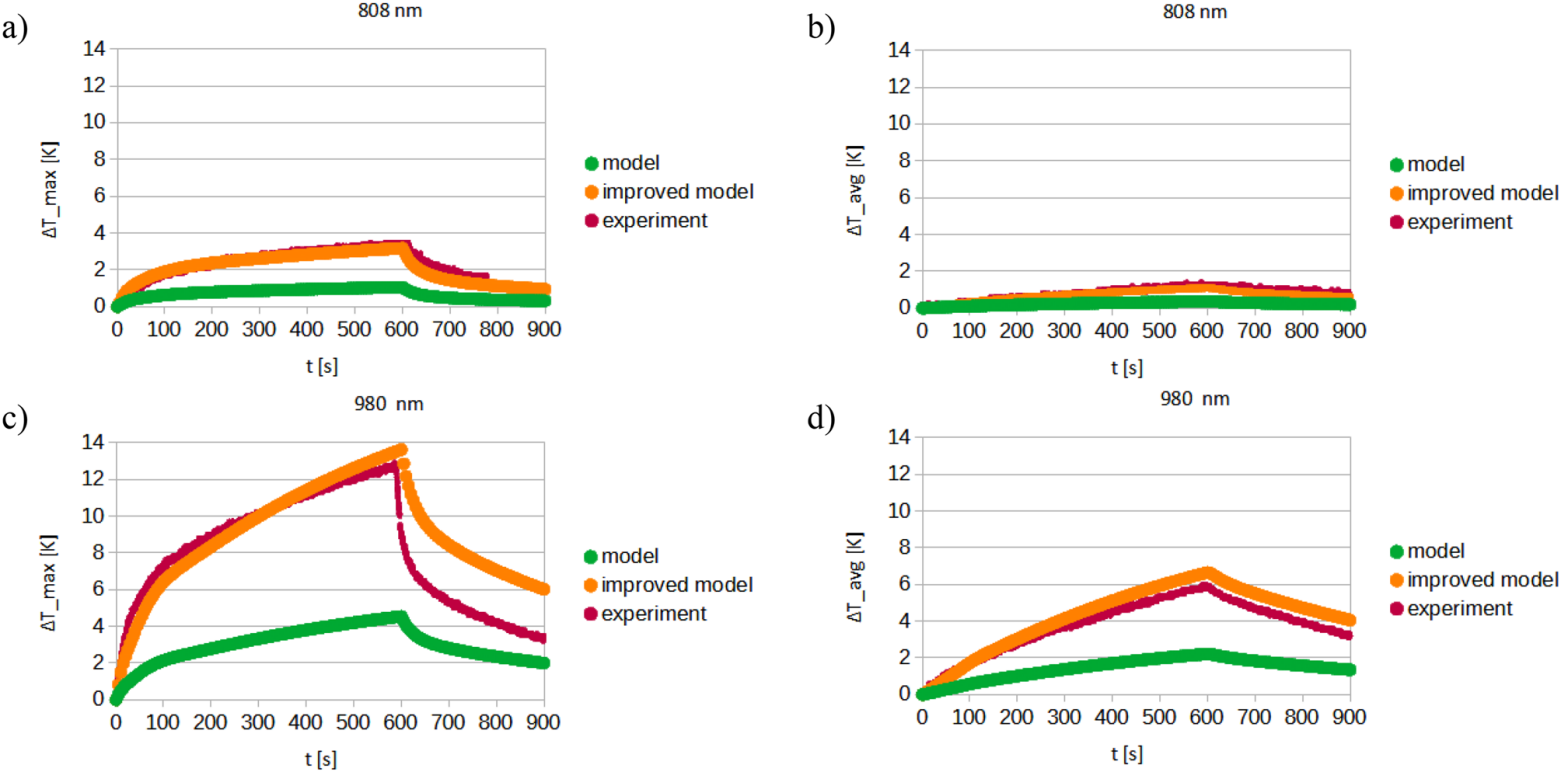
Comparison of temperature increase curves for experiment, model and model after corrections: (**a**) 808 nm laser, max. temperature, (**b**) 808 nm laser, average temperature, (**c**) 980 nm laser, max. temperature, (**d**) 980 nm laser, average temperature.

**Fig. 17:**
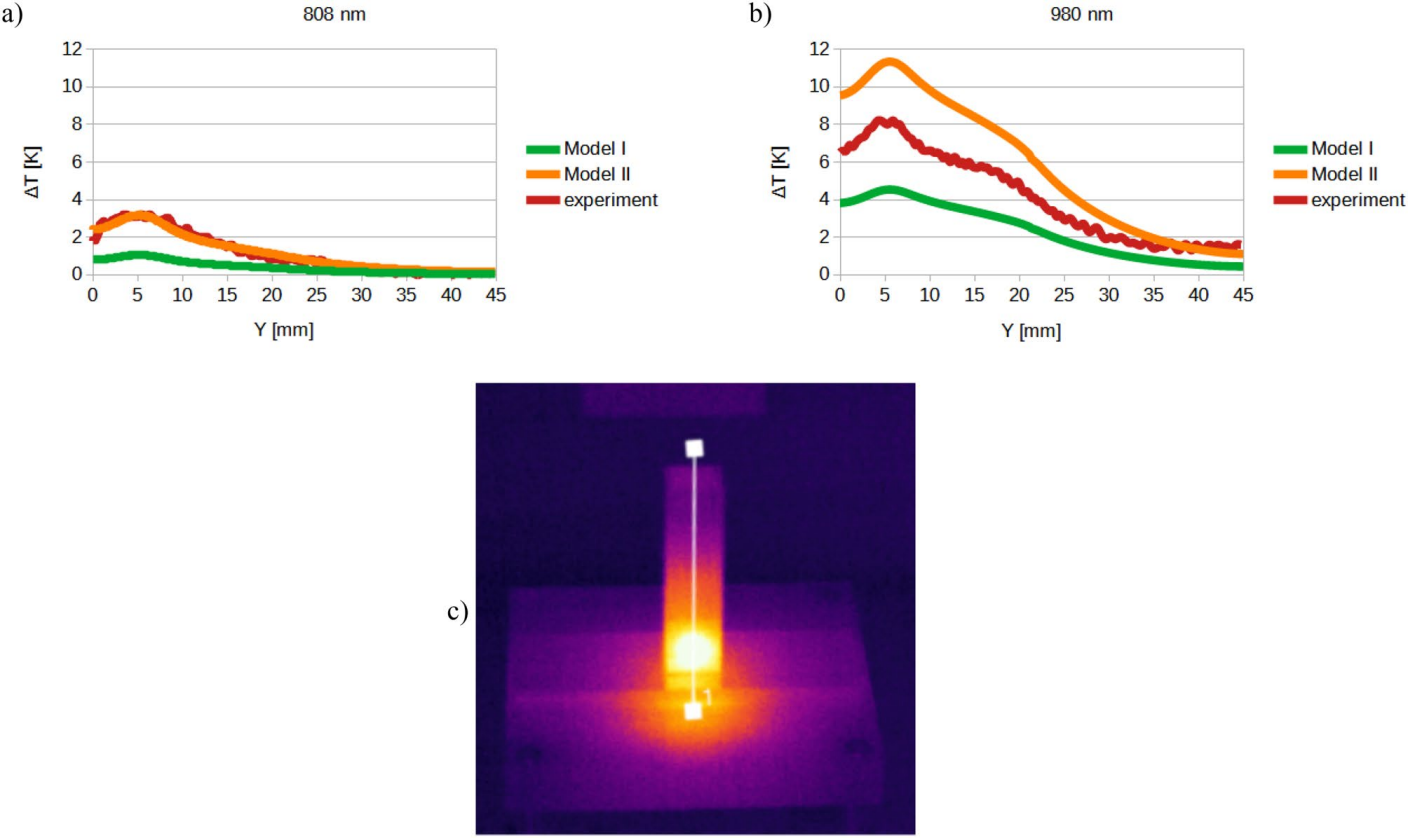
Comparison of temperature distribution on the front wall of the cuvette: (**a**) 808 nm laser, (**b**) 980 nm laser, (**c**) location of the line.

Figures 15, 16 and 17 illustrate the results of simulations conducted for laser wavelengths of 808 nm and 980 nm in the tested system before the injection of gold nanoparticles into the skin. The temperature distribution is presented for the front wall of the cuvette, the cross-section of the measurement system, and the individual tissue layers (Fig. 15). Additionally, a comparison of the obtained temperature increase curves – both average and maximum – between the computational model and the experimental data is included in Fig. 16. Furthermore, the temperature distribution curves for the front wall of the cuvette are compared to assess consistency between simulation and experiment (cf. Fig.17).

The initial simulation results were found to be approximately three times lower than the experimental values, although the general shape of the temperature curves was preserved. Following adjustments to the absorption and scattering coefficients, the simulated results aligned more closely with the experimental data. This finding underscores a fundamental challenge in modeling biological tissues – their optical and thermal properties are highly variable and influenced by numerous factors, including age, genetic background, even lifestyle. In the case of skin, additional variables such as pigmentation and anatomical location further contribute to this complexity, making the development of a universally applicable tissue model infeasible.

**Fig. 18:**
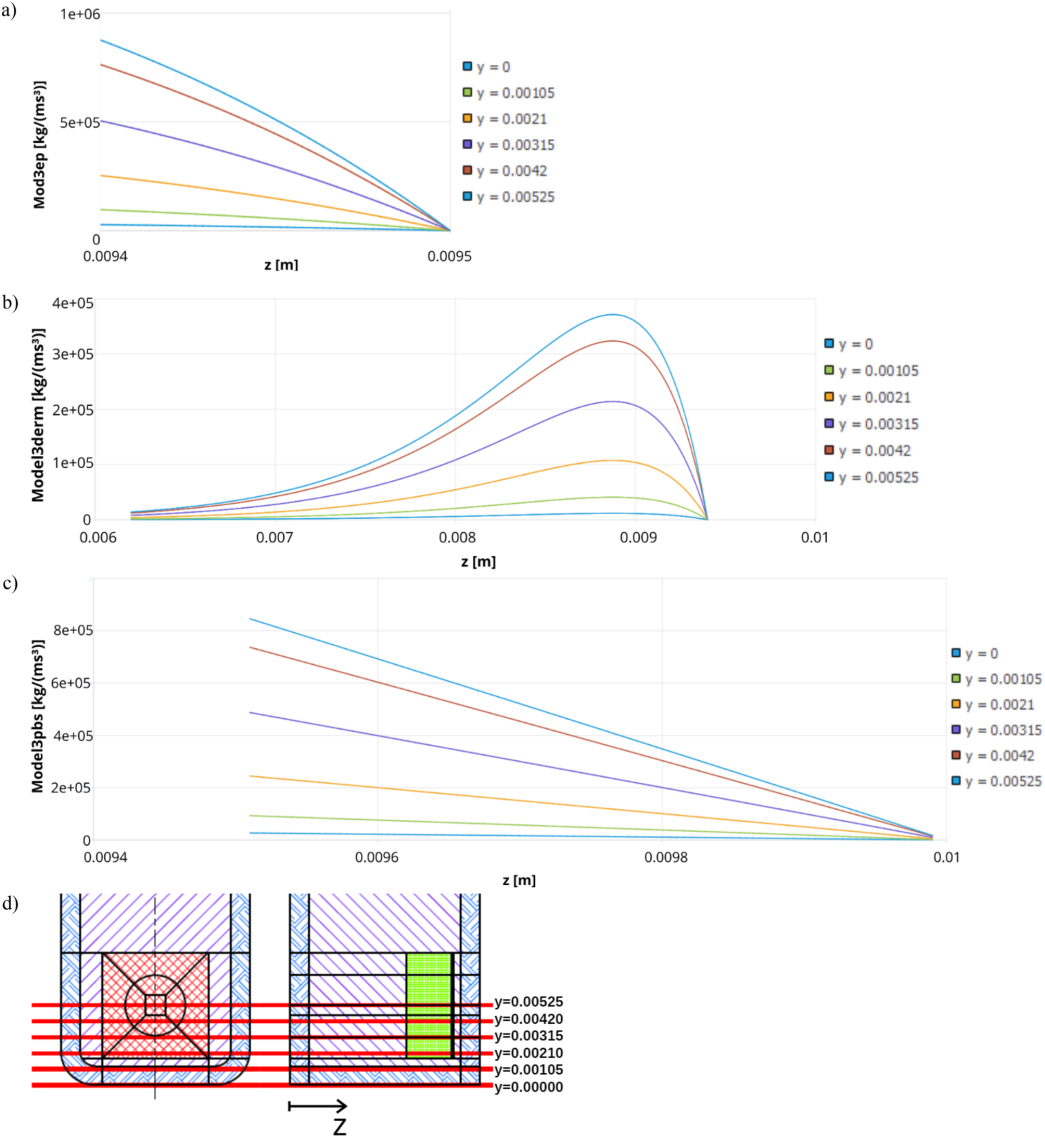
Volumetric heat generated for 980 nm laser in the tested system before the injection of gold nanoparticles into the skin in: (**a**) epidermis layer (Mod3ep), (**b**) dermis layer (Model3derm), (**c**) PBS first layer (Model3PBS), (**d**) scheme of locations of different y values.

**Fig. 19:**
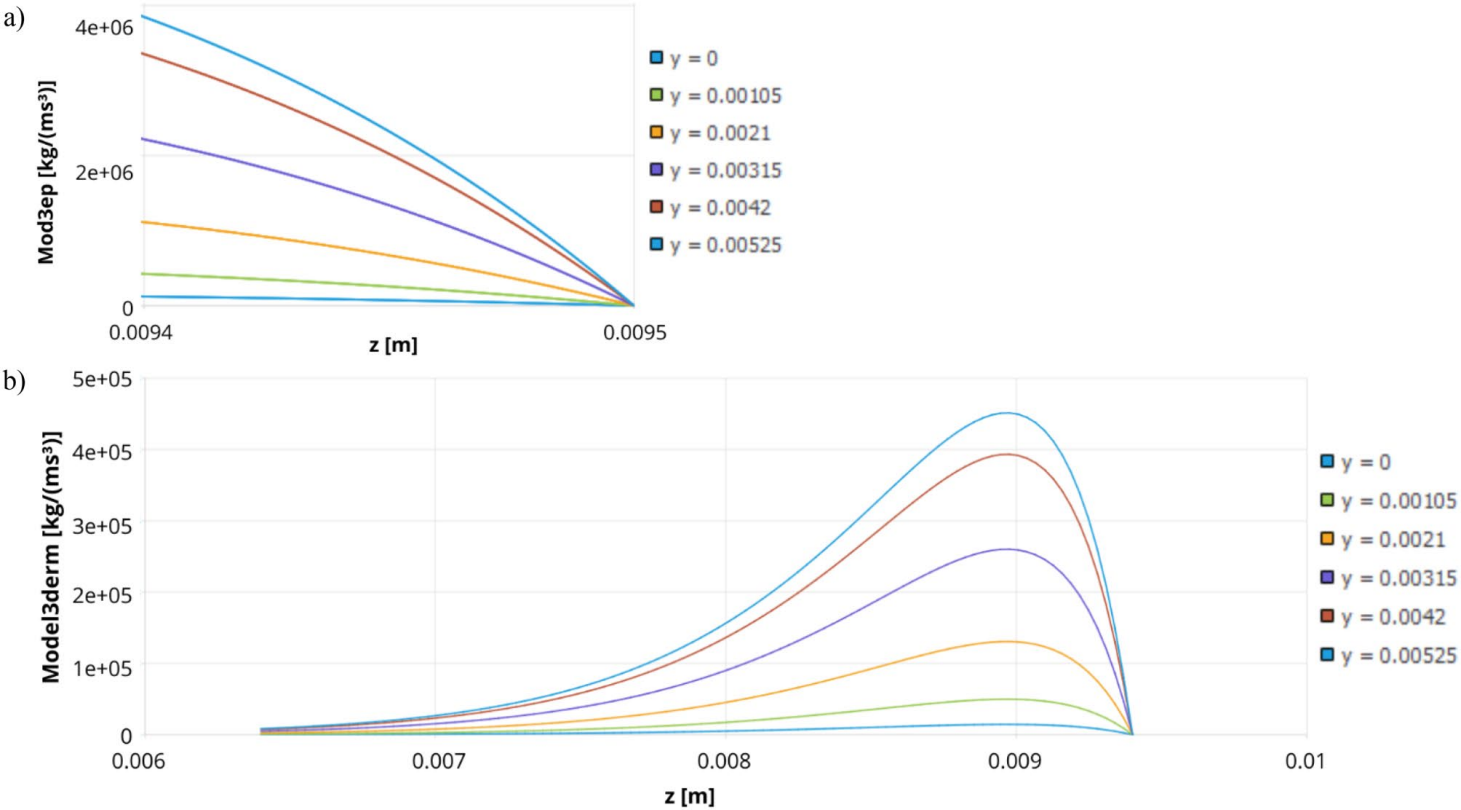
Volumetric heat generated for 808 nm laser before the injection of gold nanoparticles into the skin in: (**a**) epidermis layer (Mod3ep), (**b**) dermis layer (Model3derm).

Based on the presented formulae and results, graphs depicting the heat generation as a function of position along the z-axis were created for both the 808 nm and 980 nm laser wavelengths. The graphs were generated for a constant x-coordinate of 0.00625 m while varying the y-coordinate, ranging from the bottom of the cuvette to the midpoint of the skin-equivalent layer (Fig. 18), where the heat generation reaches its maximum value. For the 980 nm laser, heat generation also occurs within the first PBS layer, resulting in three distinct graphs for this wavelength (Fig. 18a–c). On the other hand, Fig. 19 presents volumetric heat generated for 808 nm laser in: (a) epidermis layer (Mod3ep), (b) dermis layer (Model3derm).

**Fig. 20:**
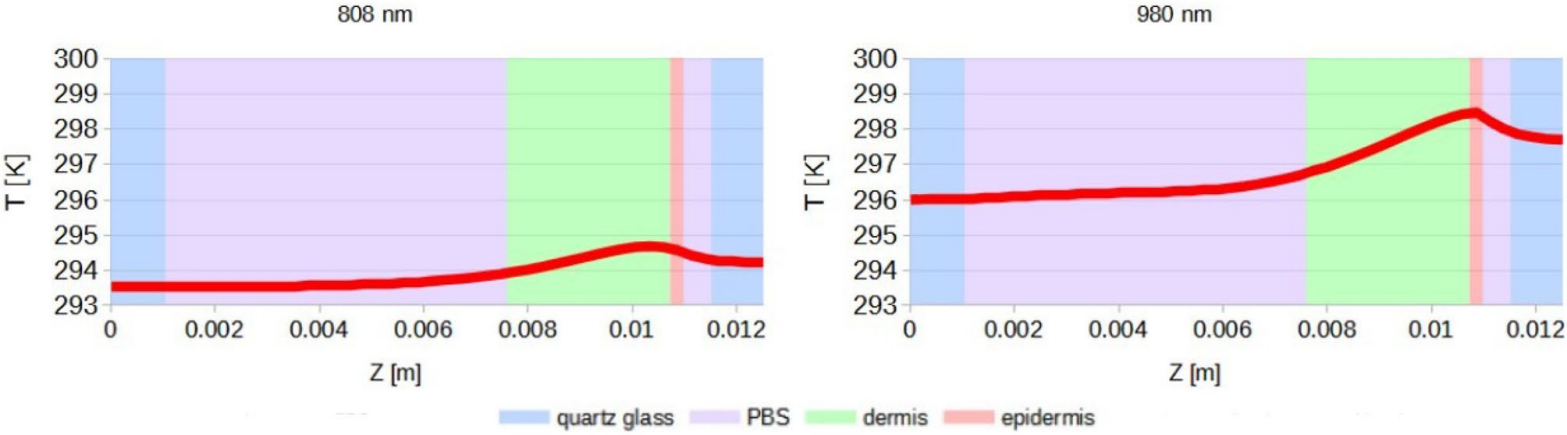
Temperature distribution in the tested system before the injection of gold nanoparticles into the skin through all model layers for x = 0.00625 m, y = 0.00525 m; time: 600 s.

Although the heat generation values do not form a continuous function between individual layers, the resulting temperature distribution across the entire system is continuous, as demonstrated in (Fig. 20).

**Fig. 21:**
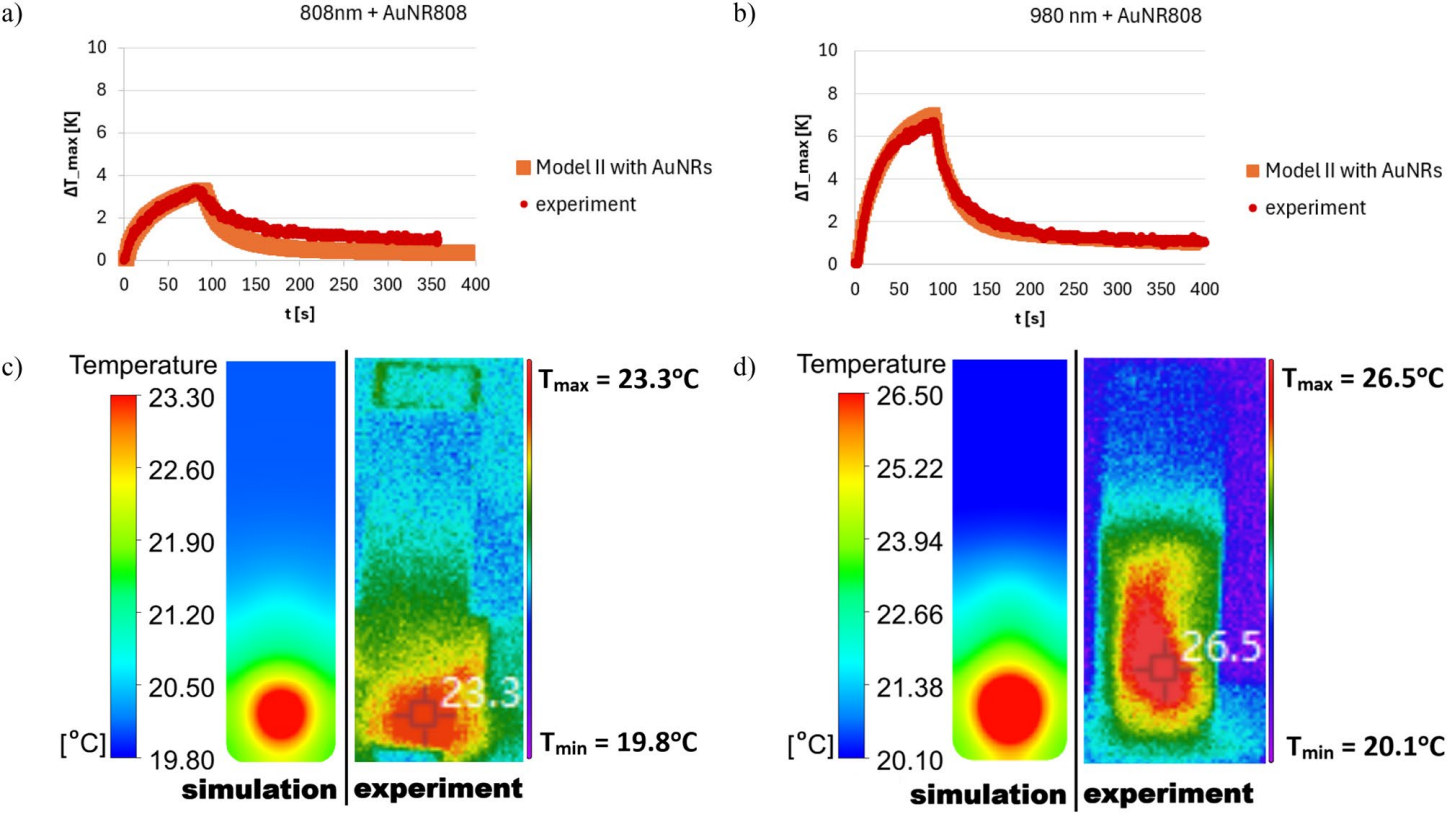
Comparison of representative results obtained from the theoretical model and experimental measurements of the temperature changes in (**a**) 808 nm and (**b**) 980 nm laser-irradiated skin with the injected AuNR808 sample; corresponding temperature distributions (**c**) wall under 808 nm and (**d**) 980 nm laser irradiation with the injected AuNR808 at the cuvette’s wall.

The particular analysis is focused on laser-irradiated AuNR808 injected into skin. Figure 21 highlights the satisfactory comparison of thermal responses and temperature distributions between theoretical modelling and experiments of laser-irradiated AuNR808 injected into skin. The convergence of the experimental data with the simulation results is particularly visible during the laser irradiation stage, where the temperature curves obtained as a result of theoretical simulations closely matches that measured by the thermal camera. During the cooling stage, a slight discrepancy can be observed, which is attributed to irregular air movements present in the laboratory environment. These fluctuations are highly unpredictable and therefore challenging to implement into numerical simulations. Moreover, for PTT applications, the laser irradiation plays a key role to establish the effectiveness of nanoparticles plasmonic heating. As such, the minor mismatches observed at the cooling phase are considered as negligible and do not affect the objectives of this study. Therefore, the presented model has been predicted to be representative at irradiation stage to estimate heat generation rate and calculate temperature changes, both for 808 and 980 nm laser wavelengths.

## Summary, conclusions and future prospects

Regarding the experimental findings, it was confirmed that NIR wavelengths - specifically 808 nm and 980 nm - are the most suitable for irradiation of human skin due to the reduced heating of human skin itself, reducing the risk of thermal damage of healthy cells, and further investigations should be focused within this spectral region.

Additionally, in contrast to laser ablation of bare skin, laser-irradiated gold nanostructures, which exhibit very high biocompatibility and biosafety, generates localized heating, which means that temperature drops fast as the distance increase to the NP surface. In this work, the results demonstrated that the injection of nanoparticles into the skin significantly enhances temperature elevation under laser irradiation compared to control samples without nanoparticles by 10–300 and 50–100% for 808-nm and 980-nm laser irradiation, respectively.

For the 808 nm laser wavelength, the highest temperature increases were observed for 5 nm diameter nanospheres after 1.5 min of irradiation and for non-commercial nanorods designated as AuNR6 after 5 min of irradiation. For the nanospheres, this result is initially surprising, as they lack absorbance at this wavelength. However, aggregation of the weakly stabilized citrate-capped nanospheres in biological tissue is indeed expected, this process leading to gold-nanoparticle clusters with red-shifted (and enhanced) plasmon resonance compared with the single nanospheres^66^. For the 980 nm wavelength, the most significant temperature increases were achieved with pegylated AuNR6 nanorods and 5 nm diameter nanospheres after 1.5 min of irradiation, as well as AuNR980 nanorods after 5 min of irradiation.

In the case of nanorods, aggregation also shifts the plasmon resonance (red- or blue-shift depending on the aggregation mode)^69^. Thus, even though the citrate-stabilized AuNR980 is resonance-matched and therefore expected to perform best, aggregation may change the situation and lead to less absorbance at the illumination wavelength. Consequently, the pegylated AuNR6, which is better stabilized but initially a little off-resonant, can in some conditions outcompete the former sample, as the AuNR6 sample is expected to be much less prone to aggregation, thus having invariant plasmonic properties also in complex media.

To conclude, these comparative results, there are thus two strategies from a fundamental perspective for optimal plasmonically-driven hyperthermia: (i) triggered aggregation of initially off-resonant gold nanoparticles (e.g. gold nanospheres) at the tumor site; and (ii) resonance engineering of gold nanorods to match the employed NIR laser wavelength with additional colloidal stabilization, e.g. by end-grafted polymer ligands. This boosts the effectiveness of tumors ablation when gold nanoparticles shall be injected directly into the tumors cells while the risk of thermal damage of healthy cells would be minimized in the biologically transparent spectral window (800–1000 nm).

However, certain inconsistencies were noted in the experimental results. In some cases, the average temperature increase following nanoparticle injection was lower than that observed in the control sample (808 nm, 1.5 min: AuNR980; 5 min: AuNSp5, AuNSp10). For the 980 nm laser, these variations were within the measurement error range. Additionally, instances were recorded where the temperature increase after 1.5 min of irradiation was higher than after 5 min (808 nm: AuNSp5, AuNSp10; 980 nm: AuNSp5, AuNSp20).

These discrepancies are most likely due to variations in the biological properties of the skin samples, which originated from different patients and, therefore, exhibited differing responses to laser irradiation. Furthermore, despite following a standardized protocol specifying sample dimensions of 7 × 7 mm, it was not always possible to precisely maintain this size, which may have influenced the results. Another potential source of variability was the influence of nanoparticles aggregation in human skin, which had shifted the peak resonance of gold nanorods significantly. Similarly, it was demonstrated that the use of relatively low concentrations of gold nanoparticles can effectively enhance the temperature increase in human skin. Moreover, there appears to be a threshold concentration beyond which further increases become unnecessary and ineffective. To address these issues, further experiments are required to improve result standardization and enhance reproducibility, ensuring greater reliability in future studies.

In fact, laser power metering allowed to establish that each wavelength that was used in these studies cannot penetrate the beam through human skin, including the near-infrared region, which has been supposed to be optically transparent. In fact, heating of bare pieces of human skin is minimum for 808 and 980 nm, the transmitted intensity, however, reaches a maximum few tenths of a percent, which constitutes a valid conclusion that open surgical operations would be essential for laser-triggered cancer therapy. Nevertheless, nanoparticles can be used both for easily accessible tumors and those that are difficult to cut with a tendency to be widely spread throughout the human body.

Furthermore, there is lack of nondestructive methods that allow to predict the temperature inside human skin. Theoretical modelling emerges here as an useful weapon to provide this issue, if the assumed model is valid. Moreover, one of the primary challenges in modeling biological tissues and structures lies in their inherent variability, as their properties are influenced by multiple factors, including age, genetic background, and tissue origin. CFD models are constrained by the idealized conditions defined within them, which may limit their ability to fully replicate biological complexity. On the other hand, theoretical modelling is supposed to discover at which types of cancers and at which conditions gold nanoparticles indicate their great potential to effective tumor overheating, which was revealed in this article. Correct determination of thermal and optical properties is crucial here to simulate the temperature distribution and to reflect the process of tumor disintegration. In this article, selected data in literature was verified in front of their validity. As long as the scattering coefficient does reflect human skin and has remained untouched in this work, the absorption coefficient was declared to be underestimated circa three times, which could be explained in the idealized properties of human skin that have been assumed following pure water properties in^53,56^.

Aiming at PTT applications, the validation of the presented model is crucial to establish the real potential of gold nanoparticles, expressed in temperature rise. The fourteen Kelvin degrees rise, assumed in this paper, is believed to activate the irreversible processes in skin. Neither 808 and 980 nm laser irradiation processes contributed to bring the effective temperature rise, which realizes to use either longer irradiation process or higher laser power sources. Here, the presented model can assist to predict the parameters, that are key to estimate the effectiveness and the cost analysis for these applications.

Future work aims to expand the model to incorporate three distinct layers, including subcutaneous tissue, followed by scaling to larger anatomical regions. This progression will facilitate the development of a more comprehensive whole-body model, enabling the optimization of therapy parameters based on the specific treatment location. Furthermore, additional refinements will include incorporating a layer representing cancerous tissue and accounting for heat generation due to nanoparticle interactions with laser irradiation.

## Data Availability

All data generated or analyzed during this study are included in this published article. The datasets used to create the figures are available from the corresponding author upon reasonable request.
