# Correction: Laser irradiation of human skin tissue after gold nanoparticles injection for thermal ablation processes – a combined experimental and numerical approach

**DOI:** 10.1038/s41598-025-32873-3

**Published:** 2025-12-29

**Authors:** Marta Cecotka, Piotr Radomski, Paweł Ziółkowski, Agata Tymińska, Katarzyna Czerwiec, Jacek Zieliński, Yue Dong, Christian Rossner, Francesca Petronella, Magdalena Narajczyk, Jakub Karczewski, Luciano De Sio, Michał Pikuła, Dariusz Mikielewicz

**Affiliations:** 1 Faculty of Mechanical Engineering and Ship Technology, Gdańsk University of Technology, Institute of Energy, Narutowicza 11/12, 80-233 Gdańsk, Poland; 2 Laboratory of Tissue Engineering and Regenerative Medicine, Division of Embryology, Department of Anatomy, Faculty of Medicine, Medical University of Gdańsk, Gdańsk, Poland; 3 Laboratory of Tissue Engineering and Regenerative Medicine, Division of Clinical Anatomy, Department of Anatomy, Faculty of Medicine, Medical University of Gdańsk, Gdańsk, Poland; 4 Department of Oncologic Surger, Medical University of Gdańsk, Gdańsk, Poland; 5 Leibniz-Institut für Polymerforschung Dresden e.V., Hohe Straße 6, 01069 Dresden, Germany; 6 Faculty of Chemistry and Food Chemistry, Technische Universität Dresden, 01069 Dresden, Germany; 7 Department of Polymers, University of Chemistry and Technology Prague, Technická 5, Prague 6, 166 28 Czech Republic; 8 National Research Council of Italy, Institute of Crystallography CNR-IC, Montelibretti Division, Area Territoriale di Ricerca di Roma 1 Strada Provinciale 35d, Montelibretti, 00010 Italy; 9 Faculty of Biology, Bioimaging Laboratory, University of Gdańsk, Gdańsk, Poland; 10 Faculty of Applied Physics and Mathematics, Gdańsk University of Technology, Institute of Nanotechnology and Materials Engineering, Narutowicza 11/12, 80-233 Gdańsk, Poland; 11 Department of Medico-Surgical Sciences and Biotechnologies, Sapienza University of Rome, Corso della Repubblica 79, 04100 Latina, Italy

Correction to: *Scientific Reports* 10.1038/s41598-025-17459-3, published online 02 October 2025

The original version of this Article contained an error in Figure 1, where the layers “DERMIS” and “EPIDERMIS” were swapped. The original Figure 1 and accompanying legend appear below.

**Fig. 1:**
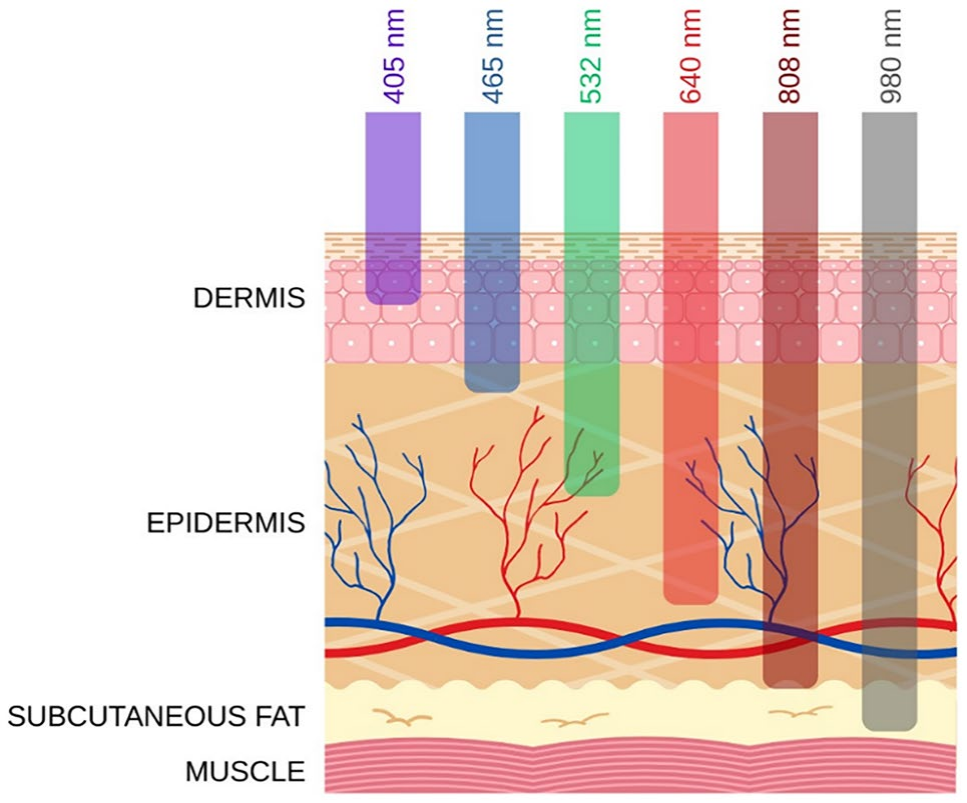
Dermal penetration of different wavelengths of light^6^.

In addition, Equation 16 displayed incorrectly. The correct equation is as follows:

$${\eta }_{PT}=1-\left(\frac{2}{3}\cdot {\left(\frac{2\pi }{\lambda }\right)}^{3}\cdot \frac{{\left({{\alpha}^{\prime}_{\mathrm{eff}}}\right)}^{2}+{(\stackrel{-}{{\alpha }_{\mathrm{eff}}})}^{2}}{\mathbbm{i}\mathbbm{m}\left({{\alpha }^{\prime}_{\mathrm{eff}}}\right)}\right)$$

The original Article has been corrected.

